# The Association of Physical Activity with Glaucoma and Related Traits in the UK Biobank

**DOI:** 10.1016/j.ophtha.2023.06.009

**Published:** 2023-06-17

**Authors:** Kian M. Madjedi, Kelsey V. Stuart, Sharon Y.L. Chua, Pradeep Y. Ramulu, Alasdair Warwick, Robert N. Luben, Zihan Sun, Mark A. Chia, Hugues Aschard, Janey L. Wiggs, Jae H. Kang, Louis R. Pasquale, Paul J. Foster, Anthony P. Khawaja

**Affiliations:** 1 NIHR Biomedical Research Centre, Moorfields Eye Hospital NHS Foundation Trust & UCL Institute of Ophthalmology, London, United Kingdom.; 2 Department of Ophthalmology, University of Calgary, Calgary, Alberta, Canada.; 3 Wilmer Eye Institute, Johns Hopkins University School of Medicine, Baltimore, Maryland.; 4 UCL Institute of Cardiovascular Science, London, United Kingdom.; 5 MRC Epidemiology Unit, University of Cambridge School of Clinical Medicine, Cambridge, United Kingdom.; 6 Department of Computational Biology, Institute Pasteur, Paris, France.; 7 Department of Ophthalmology, Harvard Medical School, Boston, Massachusetts.; 8 Brigham and Women’s Hospital / Harvard Medical School, Boston, Massachusetts.; 9 Department of Ophthalmology, Icahn School of Medicine at Mount Sinai, New York, New York.

**Keywords:** Glaucoma, Intraocular pressure, OCT, Physical activity, UK Biobank

## Abstract

**Purpose:**

To examine the association of physical activity (PA) with glaucoma and related traits, to assess whether genetic predisposition to glaucoma modified these associations, and to probe causal relationships using Mendelian randomization (MR).

**Design:**

Cross-sectional observational and gene—environment interaction analyses in the UK Biobank. Two-sample MR experiments using summary statistics from large genetic consortia.

**Participants:**

UK Biobank participants with data on self-reported or accelerometer-derived PA and intraocular pressure (IOP; n = 94 206 and n = 27 777, respectively), macular inner retinal OCT measurements (n = 36 274 and n = 9991, respectively), and glaucoma status (n = 86 803 and n = 23 556, respectively).

**Methods:**

We evaluated multivariable-adjusted associations of self-reported (International Physical Activity Questionnaire) and accelerometer-derived PA with IOP and macular inner retinal OCT parameters using linear regression and with glaucoma status using logistic regression. For all outcomes, we examined gene—PA interactions using a polygenic risk score (PRS) that combined the effects of 2673 genetic variants associated with glaucoma.

**Main Outcome Measures:**

Intraocular pressure, macular retinal nerve fiber layer (mRNFL) thickness, macular ganglion cell—inner plexiform layer (mGCIPL) thickness, and glaucoma status.

**Results:**

In multivariable-adjusted regression models, we found no association of PA level or time spent in PA with glaucoma status. Higher overall levels and greater time spent in higher levels of both self-reported and accelerometer-derived PA were associated positively with thicker mGCIPL (*P* < 0.001 for trend for each). Compared with the lowest quartile of PA, participants in the highest quartiles of accelerometer-derived moderate- and vigorous-intensity PA showed a thicker mGCIPL by +0.57 μm (*P* < 0.001) and +0.42 μm (*P* = 0.005). No association was found with mRNFL thickness. High overall level of self-reported PA was associated with a modestly higher IOP of +0.08 mmHg (*P* = 0.01), but this was not replicated in the accelerometry data. No associations were modified by a glaucoma PRS, and MR analyses did not support a causal relationship between PA and any glaucoma-related outcome.

**Conclusions:**

Higher overall PA level and greater time spent in moderate and vigorous PA were not associated with glaucoma status but were associated with thicker mGCIPL. Associations with IOP were modest and inconsistent. Despite the well-documented acute reduction in IOP after PA, we found no evidence that high levels of habitual PA are associated with glaucoma status or IOP in the general population.

**Financial Disclosure(s):**

Proprietary or commercial disclosure may be found in the Footnotes and Disclosures at the end of this article.

Physical activity (PA) is well established to be protective against various chronic diseases^1-3^ and has been associated with neuroprotective effects in age-related neurodegenerative conditions.^4,5^ Keen interest exists in whether lifestyle modifications or behaviors such as PA may affect chronic ophthalmic conditions such as glaucoma or related traits such as intraocular pressure (IOP).^6-9^ The acute, transient, IOP-lowering effects of PA in healthy people are well documented,^10-16^ with most studies reporting a modest acute IOP reduction of 1 to 5 mmHg after a period of PA. Higher intensity of PA may be associated with a greater short-term reduction in IOP,^13^ and the magnitude of change in IOP may be related to baseline fitness levels.^17^ Although many small and experimental studies have identified short-term associations with PA, fewer studies have examined the association of habitual PA with IOP, and the results are inconsistent.^18,19^

Animal studies have shown that intense exercise may protect the optic nerve from injury caused by elevated IOP and may attenuate retinal inflammatory responses.^20,21^ High levels of intense exercise in rats were associated with an increased ability to withstand retinal ganglion cell (RGC) death after acute elevations in IOP,^20^ and rats with axotomized optic nerves placed in a forced PA group showed greater RGC survival rates than rats that were not subjected to high levels of activity.^22^ These potentially neuroprotective effects of PA have been proposed to relate to cardiovascular fitness, resulting in increased perfusion to the optic nerve, retinal, and macular tissues.^23-25^ Studies of the relationship between PA and glaucoma status have reported conflicting results, with some studies demonstrating a potentially protective effect in those with high fitness levels^9,26^ and other studies finding potentially deleterious associations^27^ or no discernable association.^7,8^

Increasing evidence suggests that lifestyle factors may be evident only in patients with the highest genetic predisposition to glaucoma,^28,29^ and no studies of PA and glaucoma to date have examined potential geneeenvironment interactions. The existing literature additionally is limited by small sample sizes and the use of self-reported measures of PA.

Accelerometry has emerged as the gold standard for objective assessment of PA^30^ and is playing an increasingly important role in evaluating associations between PA and ophthalmic disease.^31-33^ Accelerometers are a validated^34^ and reliable means of capturing PA levels and allow for the objective assessment of PA, which overcomes the limitations of recall bias and heterogeneity in self-reported questionnaires.^35^ Activity typically is recorded in epochs of preprogrammed length in which activity is quantified as steps and then categorized further by the intensity of motion over each study epoch based on the amount of detected movement adjusted for body size.^35^ More recent methods involve analysis of triaxial accelerometry data obtained at subsecond resolutions to predict more accurately the amount of energy being expended,^35,36^ which then can be summarized as daily minutes spent in light, moderate, or vigorous PA.^35,37^

We conducted a large observational study evaluating associations of PA (measured using both a validated self-report questionnaire and accelerometry) with glaucoma status, IOP, and inner retinal thickness on OCT using data from the UK Biobank. We additionally examined whether genetic predisposition to glaucoma may modify any of these associations and performed 2-sample Mendelian randomization (MR) analyses to probe causal relationships.

## Methods

### UK Biobank Study Population

The UK Biobank is a large community-based cohort of more than half a million UK residents registered with the National Health Service who are 37 to 73 years of age at enrollment. Baseline examinations were carried out between 2006 and 2010 at 22 study assessment centers. The North West Multicenter Research Ethics Committee approved the study in accordance with the principles of the Declaration of Helsinki. All participants gave written informed consent before enrollment in the study. The overall study protocol and protocols for individual tests are available online (https://bio-bank.ndph.ox.ac.uk/ukb/index.cgi). Participants answered detailed touchscreen questionnaires that covered a wide range of demographic, health, and lifestyle information.^38^ We combined ethnicity groups into White and non-White, given the small proportion of non-White participants in the UK Biobank cohort. The Townsend deprivation index was determined according to the participants’ postcode at recruitment and the corresponding output area from the preceding national census. The index was calculated based on the output area’s employment status, home and car ownership, and household condition: the higher and more positive the index, the more deprived the area. Smoking and alcohol intake status were determined by self-report and were categorized into never, former, or current use.

Diabetes status was defined by self-report of diabetes mellitus or use of antidiabetic medications. Systolic blood pressure was measured twice using the HEM-70151T digital blood pressure monitor (OMRON), and the mean was used in the analysis. Weight was measured with the BV-418 MA body composition analyzer (Tanita). Height was measured using a Seca 202 stadiometer (Seca). Body mass index was calculated as weight (in kilograms) per height (in square meters).

### Assessment of Physical Activity Measurements

Baseline assessment included information on self-reported PA using an adapted version of the validated International Physical Activity Questionnaire (IPAQ),^39^ which was completed on a tablet computer. Participants were asked how many days per week they participated in ≥ 10 minutes of each of the following types of PA: sedentary (e.g., sitting, driving, and watching television), light PA (e.g., walking), moderate PA (e.g., carrying light loads and bicycling at a regular pace), and vigorous PA (e.g., heavy lifting, digging, and aerobics or fast bicycling) in a typical week and then were asked to report how many minutes they participated in each of these activity levels on a typical day.

International Physical Activity Questionnaire PA data then were processed in line with the IPAQ guidelines.^40^ Metabolic equivalents of task (METs), an objective measurement of the ratio of energy expenditure rate to an individual’s mass, were calculated and scored as 2.3, 3.0, and 7.0 METs for light, moderate, and vigorous PA, respectively. A composite score of overall PA level also was determined (categorized into low, moderate, and high). Participants’ average METs per week were calculated by taking the time spent in each of these activities reported on a typical day multiplied by the typical number of days the exercise was reported and the respective MET scores for that type of activity.

Accelerometry-derived PA was measured in a subset of participants in the UK Biobank. Between February 2013 and December 2015, approximately 100 000 participants were invited to wear a commercial triaxial accelerometer on the wrist of their dominant arm (Axivity AX3; Axivity Ltd.) continuously for 7 days.^35^ Axivity AX3 records acceleration data in 3 axes (x-axis, y-axis, and z-axis) at a frequency of 100 Hz and acceleration range of ±8 g. Raw accelerometer data were collected after accelerometers were returned by mail and were calibrated,^41^ and wear times were identified using the UK Biobank preprocessing methods described previously.^35^ Raw accelerometer data were transformed into summary measurements over 5-second epochs (maintaining the average vector magnitude over the epoch),^35^ providing total mean acceleration over the 7-day measurement period, mean hourly acceleration, and time spent within a range of different mean acceleration values as a marker of PA intensity. The proportion of time spent in sedentary, light, moderate, and vigorous PAs was defined as the proportion of time spent in accelerations of ≤ 25 milligravity, 26 to 100 milligravity, 101 to 425 milligravity, and ≥ 425 milligravity, respectively.^42^ Participants with poor wear times or with data that could not be calibrated (> 1% of clips [values of > 8 g or < 8 g] or abnormal average acceleration [> 100 mg]) were excluded from the analysis.

### Intraocular Pressure Measurement

Ocular assessment was introduced as an enhancement in 2009 and 2010. Ophthalmic data were collected for approximately 115 000 UK Biobank participants at 6 assessment centers across the United Kingdom. Intraocular pressure was measured once for each eye using the Ocular Response Analyzer noncontact tonometer (Reichert Corp.).^43^ Participants who had undergone eye surgery within the previous 4 weeks or those with an eye infection were excluded from undergoing IOP measurement. The Ocular Response Analyzer flattens the cornea with a jet of air, causing an initial inward applanation, followed by an outward applanation event as the cornea returns to its original shape. An electro-optical system measures the air pressures at the initial inward applanation and the outward applanation event and combines them linearly to derive an IOP that accounts for corneal biomechanical properties.^44^ This corneal-compensated IOP was used as the value for IOP in our analyses because corneal-compensated IOP may be more associated closely with glaucoma progression and is less influenced by corneal biomechanical properties.^45^

Participants who previously had received glaucoma laser therapy or had undergone glaucoma surgery were excluded from analyses with IOP because of the impact of glaucoma treatment on IOP. A likely substantial proportion of participants with high IOPs in the cohort were treated with IOP-lowering medication in the community before entering the study (and pretreatment IOP was not available). Therefore, we imputed pretreatment IOP by dividing the measured IOP by 0.7 in participants reporting current use of IOP-lowering medication to account for mean IOP reduction achieved by medication. This method has been used previously in published genetic and epidemiologic studies of IOP.^29,46^

Participant-level IOP was calculated as the average of both eyes or as either right or left eye value if data were available for only 1 eye. Spherical equivalent was calculated for each participant and was included in our multivariable models because refractive error may influence both inner retinal thickness measurements (via magnification artefacts) and the tendency to engage in PA.

### OCT Data

As part of the UK Biobank Eye and Vision Consortium, approximately 65 000 individuals underwent macular spectral-domain OCT imaging as part of baseline examinations between 2009 and 2010.^47^ The Topcon 3D OCT1000 Mark II was used to complete spectral-domain OCT imaging in a dark room without pupil dilation. The 3-dimensional 6 × 6—mm^2^ macular volume scan mode (512 A scans per B scan and 128 horizontal B scans in a raster pattern) was used for imaging. Both eyes were imaged starting with the right eye. Details of the data acquisition and quality control are described in Appendix 1 (available at www.aaojournal.org). We used average thickness parameters derived from the macula 6 grid. Participant-level macular retinal nerve fiber layer (mRNFL) and macular ganglion cell—inner plexiform layer (mGCIPL) thicknesses (in micrometers) were calculated as the mean of right and left eye values for each participant with good-quality images available for both eyes. If data were available only for 1 eye, we considered that value for the participant.^47^ Peripapillary RNFL thickness was not measured in the UK Biobank.

### Ascertainment of Glaucoma Status

From 2006 through 2010, the touchscreen questionnaire administered to approximately 175 000 participants included a question on physician-diagnosed eye disorders. Participants were considered cases if they reported a diagnosis of glaucoma or a history of glaucoma surgery or laser therapy in either eye. We also included any participant carrying an International Classification of Diseases (ICD) code for glaucoma (ICD, Ninth Revision: 365.* [excluding 365.0]; ICD, Tenth Revision: H40.* [excluding H40.0 and H42.*]) in the linked hospital records at any point before and up to 1 year after the baseline assessment. We excluded patients who received a diagnosis before 30 years of age and control participants who reported using ocular hypotensive medication or carrying an ICD code for glaucoma suspect (ICD, Ninth Revision: 365.0; ICD, Tenth Revision: H40.0).

### Genotyping Data and Glaucoma Multitrait Analysis of Genome-Wide Association Study Polygenic Risk Score

Genetic data on approximately 490 000 UK Biobank participants were generated using 2 genotyping arrays. The Affymetrix UK BiLEVE Axiom Array^48^ returned genotypes at 807 411 markers on approximately 50 000 individuals polygenic risk score, and the Affymetrix UK Biobank Axiom Array generated genotypes at 825 925 markers for the remaining 450 000 individuals. Because these platforms share 95% of genetic markers, quality controls and imputation (the determination of genotypes at loci by inference and not by direct genotyping) were performed jointly, as described previously.^49^ In particular, imputation was based on genetic architecture ascertained in the UK 10K and the Haplotype Reference Consortium reference panels. After quality control, 92 693 895 genetic markers of 487 442 participants were available.

For glaucoma-related genetic variants, we constructed a polygenic risk score (PRS) based on 2673 independent single nucleotide polymorphisms (SNPs) associated with glaucoma (*P* ≤ 0.001) from a recent multitrait analysis of a genome-wide association study (GWAS) that included the UK Biobank.^50^ Glaucoma is a complex disease, and we considered the multitrait analysis of a GWAS PRS to be a better representation of genetic variation in glaucoma than any individual or limited set of variants. We used the effect estimates from the original multitrait analysis of a GWAS to generate a glaucoma PRS for each participant using a standard weighted sum of individual SNPs:

$$\sum_{i=1}^{2673}{\hat{\beta }}_{\left(i\right)}*{SNP}_{\left(i\right)},$$

where ${\hat{\beta }}_{\left(i\right)}$ is the estimated effect size of ${\text{SNP}}_{\left(i\right)}$ on glaucoma. The PRS was normalized with a mean of 0 and standard deviation of 1 for analyses. The PRS was included as a continuous explanatory variable in the maximally adjusted linear and logistic regression models with the addition of an interaction term between the PRS and physical activity. This model therefore allows for an assessment of the associations with both PRS (gene) and physical activity (environment), as well as the interaction of the two (gene—environment association).

### Statistical Analyses

The baseline characteristics of participants were determined and presented as mean ± standard deviation for continuous variables and number (percentage) for categorical variables. We used multivariable linear regression to examine associations between PA with IOP and OCT parameters and logistic regression to assess associations with glaucoma status. Analyses were adjusted for age, sex, ethnicity (White and non-White), smoking status (never, former, and current use), alcohol intake (never, former, and current use), Townsend deprivation index (range, −6 to 11), body mass index (kg/m^2^), systolic blood pressure (in millimeters of mercury), self-reported history of diabetes (yes or no), spherical equivalent (diopters; calculated as sphere power plus one-half cylinder power), and height (in centimeters), whereas analyses using accelerometer-derived PA additionally were adjusted for season (spring, autumn, winter, and summer).

Overall, IPAQ PA level was classified into low, moderate, and high PA based on MET minutes per week, and these categories were assessed further as quartiles within each PA level (quartiles 1—4, with quartile 1 representing the lowest quartile of PA in each level). We also assessed associations with IPAQ PA as a continuous variable (per 30-MET minutes per week increase in time spent within given activity level). Accelerometer-derived PA was classified into time spent in sedentary, light intensity, moderate intensity, and vigorous intensity activity. Each of these was assessed as a quartile of time spent within a given activity level and as a continuous variable (per additional 30 minutes spent per week in a given level of activity).

Statistical analyses were conducted using STATA version 15.1 software (StataCorp). We conducted the following sensitivity analyses: (1) additionally adjusting for caffeine intake given previously demonstrated associations with glaucoma and PA; (2) restricting analyses with OCT parameters to participants without neurologic conditions (specifically, Alzheimer’s disease, Parkinson’s disease, and multiple sclerosis) based on hospital episode statistics, self-report, and death certificate assessment; and (3) additionally adjusting for glycated hemoglobin (HbA1c) in analyses of inner retinal thickness, given the known potential effect of these conditions on inner retinal thickness.

### Mendelian Randomization Analyses

We conducted 2-sample MR analyses to test for a causal association between 2 genetically determined PA phenotypes and 5 glaucoma-related outcomes. We used published data from a recent large GWAS meta-analysis of physical activity^51^ to construct instrumental variables for leisure screen time (LST) and moderate to vigorous physical activity (MVPA). We included only significant SNPs from the primary meta-analyses of European ancestry participants (up to 606 820 for LST and 526 725 for MVPA). We used summary statistics from large GWAS and meta-analyses of GWAS for 5 glaucoma-related outcomes that included IOP (n = 139 555),^46^ mRNFL (n = 31 434),^52^ mGCIPL (n = 31 434),^52^ vertical cup-to-disc ratio (n = 111 724),^53^ and primary open-angle glaucoma (n = 216 257).^54^ Primary analyses were performed using a random-effects inverse-variance weighted method,^55^ with the weighted median,^56^ MR-Egger,^57^ and MR pleiotropy residual sum and outlier^58^ methods used as sensitivity analyses. Full details of the MR analyses are available in Appendix 2 (available at www.aaojournal.org).

## Results

**Figure 1.**
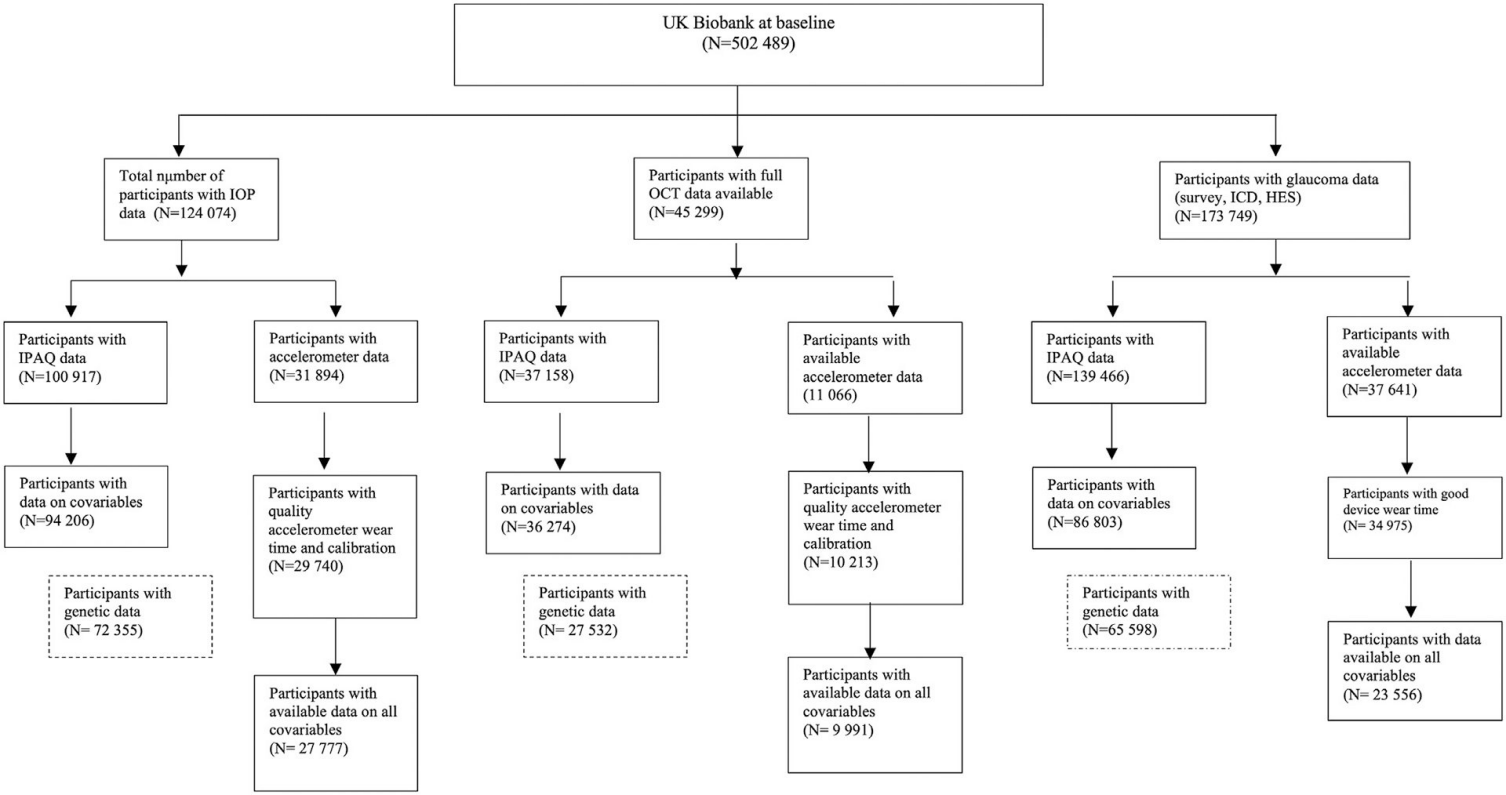
Flow diagram outlining eligible UK Biobank participants available for this study. HES = Hospital Episode Statistics; ICD = International Classification of Diseases; IOP = intraocular pressure; IPAQ = International Physical Activity Questionnaire; PCA = principal components analysis.

The sample sizes and derivation of eligible UK Biobank participants with complete data for our analyses are presented in Figure 1. Baseline characteristics of each subpopulation in our analysis are presented in Table 1.

### Associations with Glaucoma

For associations with glaucoma status, we included 86 803 participants with IPAQ PA data (baseline age, 56.6 ± 8.1 years) and 23 556 participants with accelerometer-derived PA (baseline age, 56.5 ± 7.9 years). No significant association was identified between any measure of PA and glaucoma status (Table 2).

### Associations with Intraocular Pressure

For associations with IOP, we included 94 206 participants with IPAQ PA data (average age, 56.5 ± 8.0 years) and 27 777 participants (average age, 56.5 ± 7.8 years) with accelerometry-derived PA. In a maximally adjusted multivariable model, self-reported overall habitual moderate and high PA levels were associated with a very modestly higher IOP compared with participants who reported the lowest level of overall PA (difference in IOP: +0.06 mmHg [95% confidence interval [CI], 0.00—0.12 mmHg; *P* = 0.037] for moderate and +0.08 mmHg [95% CI, 0.02—0.14 mmHg; *P* = 0.010] for high) with an overall significant trend for higher habitual overall PA associated with very modestly higher IOP (*P* = 0.017 for trend; Table 3). Associations with accelerometer-derived PA and IOP identified a very modestly lower IOP in for each additional 30 minutes spent per week in light PA, but no associations were identified with additional time spent in any other level of PA intensity. Notably, a sensitivity analysis examining the association between PA and IOP in participants with ocular hypertension (defined as an IOP > 21 mmHg) identified a very modestly lower IOP for each additional 30 MET minutes spent per week in vigorous PA (−0.002 mmHg; 95% CI, −0.003 to —0.002 mmHg; *P* = 0.022) in the IPAQ analyses, although this was not replicated in the accelerometry data. This is likely related to differences in statistical power across groups. Given that glaucoma medications may affect exercise tolerance, we carried out an additional sensitivity analysis excluding all participants using glaucoma medications and found no material difference in the identified associations in both groups.

### Associations with Inner Retinal Thickness

**Figure 2.**
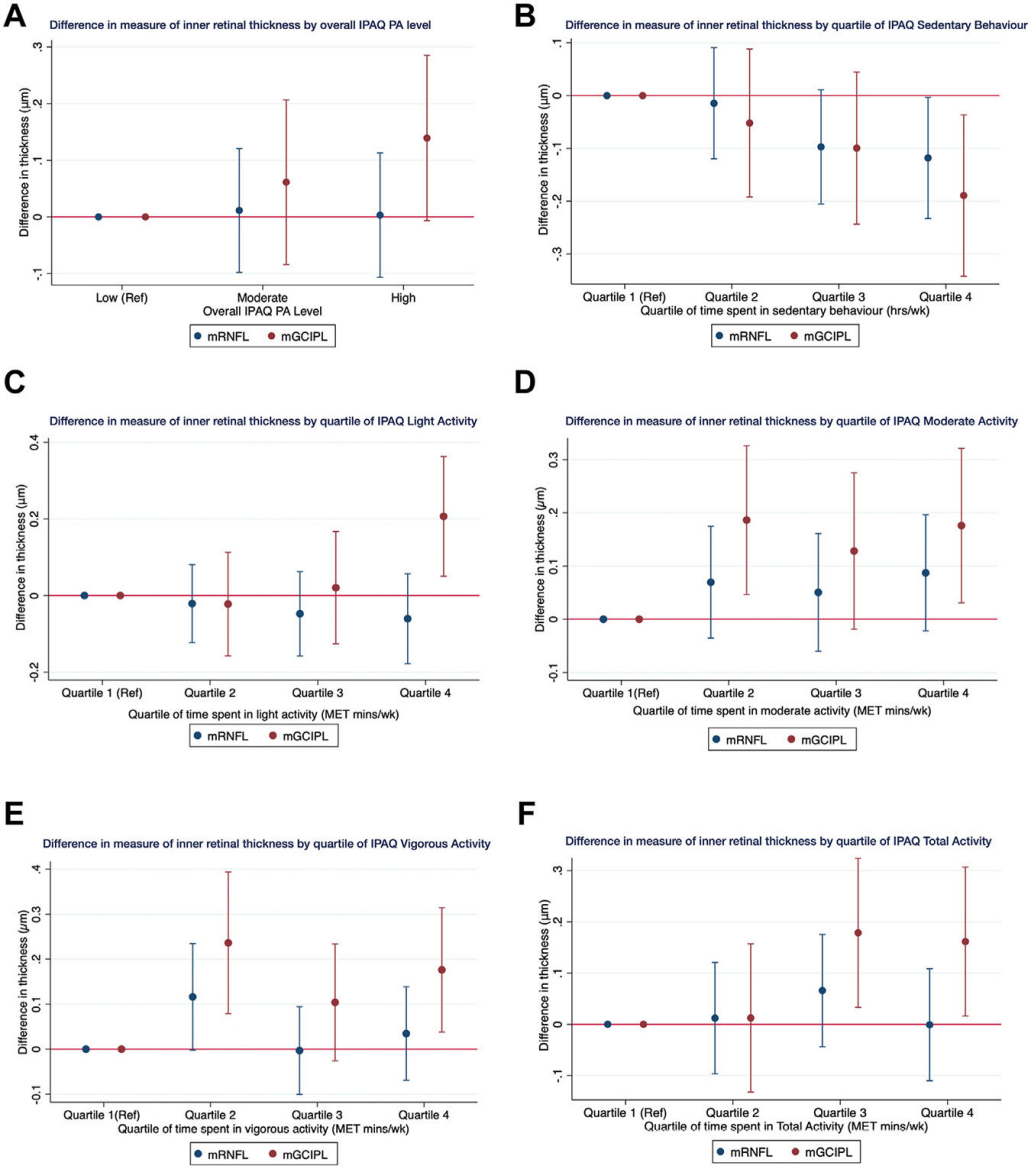
Graphs showing difference in measures of inner macular thickness by quartile of time spent in various levels of self-reported physical activity using the International Physical Activity Questionnaire (IPAQ): (**A**) overall IPAQ physical activity (PA) level, (**B**) by quartile of IPAQ sedentary behavior, (**C**) by quartile of IPAQ light activity, (**D**) by quartile of IPAQ moderate activity, (**E**) by quartile of IPAQ vigorous activity, and (**F**) by quartile of IPAQ total activity. MET = metabolic equivalent of task; mGCIPL = macular ganglion cell—inner plexiform layer; mRNFL = macular retinal nerve fiber layer; Ref = reference.

For associations with OCT measurements of inner retinal thickness (mRNFL and mGCIPL), we included 36 274 participants with IPAQ PA (average age, 56.2 ± 8.2 years) and 9991 participants with accelerometer-derived PA (average age, 56.4 ± 7.9 years). International Physical Activity Questionnaire PA was not associated with mRNFL thickness (*P* = 0.99 for trend; Table S4, available at www.aaojournal.org). Higher levels of self-reported PA were associated with a thicker mGCIPL (*P* < 0.001 for trend; Table S5, available at www.aaojournal.org). Each additional 30 MET minutes spent in total IPAQ PA was associated with a modestly thicker mGCIPL (measured in micrometers; Table S5). These associations were maintained when examining the difference in mGCIPL thickness across quartiles of time spent in IPAQ PA levels (Fig 2A-F). Compared with participants in the lowest quartiles, those in the highest quartile of self-reported time spent in each of light, moderate, and vigorous PA showed thicker mGCIPL by +0.03 μm (95% CI, 0.05—0.36 μm; *P* = 0.009), +0.18 μm (95% CI, 0.03—0.32 μm; *P* = 0.018), and +0.18 μm (95% CI, 0.04—0.31; *P* = 0.012), respectively. A similar positive association was identified between increasing quartile of time spent in total PA and thicker mGCIPL: participants in the second highest and highest quartile (i.e., quartiles 3 and 4) showed thicker mGCIPL by +0.18 μm (*P* = 0.016) and +0.16 μm (*P* = 0.029), respectively, compared with those in the lowest quartile, with a significant trend (*P* = 0.005 for trend).

**Figure 3.**
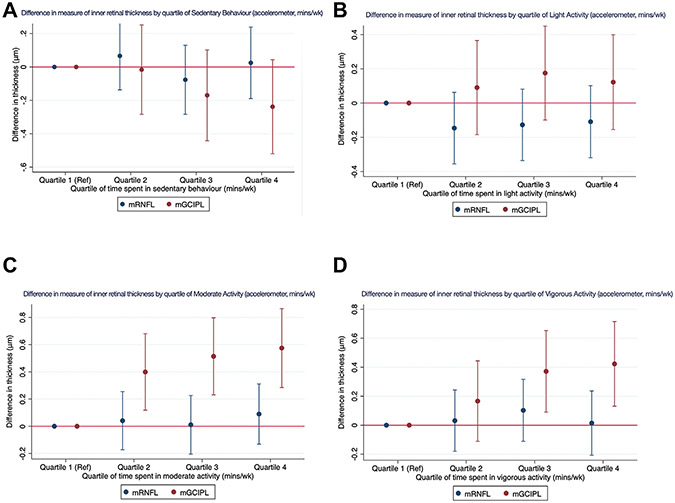
Graphs showing difference in OCT parameter thickness by quartile of time spent in various levels of accelerometry-derived physical activity (PA): (**A**) by quartile of sedentary behavior (accelerometer, minutes per week), (**B**) by quartile of light activity (accelerometer, minutes per week), (**C**) by quartile of moderate activity (accelerometer, minutes per week), and (**D**) by quartile of vigorous activity (accelerometer, minutes per week). mGCIPL = macular ganglion cell—inner plexiform layer; mRNFL = macular retinal nerve fiber layer; Ref = reference.

Analyses with accelerometry-measured PA levels identified similar associations with mGCIPL: increasing duration of time spent in moderate and vigorous PA was associated with increased mGCIPL thickness. For each additional 30 MET minutes spent in moderate PA, mGCIPL was thicker by +0.02 μm (95% CI, 0.01–0.03 μm; *P* < 0.001) and by +0.11 μm (95% CI, 0.03–0.18; *P* = 0.005), and these associations were maintained when examining the difference in mGCIPL thickness across quartiles of PA intensity levels (Fig 3A-D; Table S5). Macular ganglion cell–inner plexiform layer was approximately 0.5 μm thicker among participants in the highest quartiles of moderate (+0.57 μm; 95% CI, 0.28–0.87 μm; *P* < 0.001) and vigorous (+0.42 μm; 95% CI, 0.13–0.72 μm; *P* = 0.005) PA, with significant trends (*P* < 0.001 for trend and *P* = 0.002, respectively).

Sensitivity analyses additionally adjusting for caffeine (Table S6, available at www.aaojournal.org) and excluding participants with Alzheimer’s disease, Parkinson’s disease, or multiple sclerosis (Table S7, available at www.aaojournal.org) resulted in very modest attenuation of the associations with mGCIPL, although they all remained nominally significant. Additional adjustment for HbA1c level in analyses with mGCIPL thickness did not result in any changes in the magnitude or direction of the identified associations (Table S8, available at www.aaojournal.org).

### Genetic Modification of the Association of Physical Activity with Intraocular Pressure, OCT Parameters, and Glaucoma Status

We examined whether the association of PA with glaucoma and related traits differs based on genetic propensity for glaucoma. These analyses were restricted to genetically European participants based on principal components analysis. We included 65 598 participants with data on glaucoma status, 27 532 participants with data on mRNFL and mGCIPL thickness, and 72 355 participants with data on IOP. No evidence was found for effect modification of the multitrait analysis of a GWAS PRS on the associations between physical activity and glaucoma status (*P* = 0.07 for interaction), mRNFL thickness (*P* = 0.34 for interaction), mGCIPL thickness (*P* = 0.87 for interaction), and IOP (*P* = 0.57 for interaction).

### Mendelian Randomization Analyses

The primary MR analyses did not support a causal association between LST and any glaucoma-related outcome (*P* > 0.12 for all), with similar null associations for all sensitivity analyses. A suggestive association (not meeting the Bonferroni-corrected significance threshold) was found between MVPA and lower IOP (*P* = 0.014). Although this finding was supported by both the weighted median and MR-Egger methods, evidence was found for significant directional pleiotropy (*P* = 0.016, MR-Egger intercept test). Similarly, significant associations between MVPA and primary open-angle glaucoma for the weighted median and MR-Egger methods were marked by significant global heterogeneity (*P* < 0.001) and directional pleiotropy (*P* = 0.046), suggesting a violation of the exclusion restriction (third instrumental variable) assumption. Full results of the MR analyses are available in Appendix 2.

## Discussion

In this large study of UK Biobank participants, we observed that higher overall habitual levels of PA and greater duration of time spent in PA was not associated with glaucoma status but was associated positively with thicker mGCIPL and very modestly higher IOP. We did not identify any association between PA and mRNFL thickness, and none of the identified associations were modified by genetic predisposition to glaucoma. Furthermore, MR analyses did not support a causal association between LST and any glaucoma-related outcome. The suggested association between MVPA and lower IOP showed directional pleiotropy, suggesting that the association with IOP is not mediated through PA.

The relationship between PA and glaucoma status has been examined previously with conflicting results.^7,8,27^ Our analyses do not support an association between PA and glaucoma in the UK Biobank. Our case ascertainment criteria purposefully were broad (including self-reported diagnosis of glaucoma, history of glaucoma surgery or laser treatment, and any participant with an ICD code for glaucoma, as well as linked hospital records at any point before and up to 1 year after baseline UK Biobank assessment).

The existing studies on PA level and IOP generally have been conducted in smaller sample sizes and largely have used self-reported measures of PA. Most studies report a modest acute IOP-lowering effect of 1 to 5 mmHg after a period of PA,^10-16^ with the magnitude of IOP change potentially relating to activity intensity^59^ or baseline health status.^17^ Very little prior research has examined the association of IOP with habitual levels of PA^18,19^ or has assessed the association using more objective measures such as accelerometry. Our study found that habitual overall PA levels may be associated with a very modestly higher IOP (after adjustment for demographic, medication, and lifestyle factors), although we did not identify a dose-response association. This potentially suggests that no biological association may exist between PA and IOP and that the well-documented short-term reduction in IOP after a period of PA may be transient and may not translate into longer-term lower IOP. These associations were maintained after adjustment for both age and age squared in multivariable analyses, given that exercise level and intensity may differ based on age. Another possibility, based on animal studies, is that the level of PA necessary to influence IOP in any way is particularly high and PA levels in the UK Biobank may be too low to observe an effect.

Sensitivity analyses using deciles of PA did not show any differences in magnitude, direction, or significance of the association with IOP, although a sensitivity analysis limited to participants with IOP of > 21 mmHg found a modestly lower IOP with increasing time spent in vigorous PA. This may suggest that a beneficial effect of exercise on IOP may be apparent only in people with high IOP. Given the post hoc nature of this analysis, further replication in an independent study would be valuable to test this hypothesis further.

Because early glaucoma can affect inner retinal structures in the macula, OCT assessment of the macular region is helpful in diagnosing glaucoma.^48,60,61^ We assessed the association with mRNFL and mGCIPL and found higher levels of PA to be associated with thicker mGCIPL. We identified a clear dose-response association, with a higher overall level of self-reported PA associated with thicker mGCIPL, and this trend similarly was demonstrated within increasing quartiles of reported time spent at given levels of higher activity intensity. These associations were supported further by accelerometry analyses that also found strong associations with greater time spent in more high PA intensities and thicker mGCIPL. Macular ganglion cell—inner plexiform layer thickness is altered in diabetes, and, given the strong protective effect of PA on diabetes, residual confounding by diabetes (or related traits such as glycated hemoglobin) was considered as a possible explanation for our findings. In sensitivity analyses with additional adjustment for HbA1c (in addition to diabetes status), the associations between higher PA and thicker mGCIPL were maintained, suggesting that diabetes status and HbA1c may not necessarily explain these associations. These findings collectively support the notion that habitual PA may be associated with a thicker mGCIPL in the general population.

This association with mGCIPL thickness has biological plausibility because strong evidence exists supporting a potentially neuroprotective role of PA^4,62,63^ and experimental studies suggest that this protection also may extend to RGCs.^20,22^ Proposed mechanisms for a neuroprotective effect of PA on RGCs include an increase in retinal tissue perfusion,^24,64^ the inhibition of complement-mediated pathways,^65-68^ and an increase in neurotrophic factors.^21^ Complement dysregulation has been proposed as one potential mechanism contributing in part to the glaucomatous process,^65-68^ and PA in animal studies of induced optic nerve injury was found to block synaptic complement deposition.^21^ Aberrant neurotrophic factor expression also has been proposed as a contributory mechanism to glaucoma, and PA is associated with the upregulation of neurotrophic factors relevant to neuronal integrity. For example, brain-derived neurotrophic factor, an important neurotrophic factor involved in the differentiation of RGCs, is increased in exercise,^69-71^ and deficient brain-derived neurotrophic factor expression has been linked to RGC death^72^ in experimental glaucoma.^72-76^

This study had several strengths. This was a large study that examined multiple glaucoma-related traits and adjusted for a wide array of lifestyle, demographic, and anthropometric covariables. This allowed for a well-powered, broad investigation of the association with PA while minimizing potential residual confounding. By using two independent measures of PA (self-report and accelerometry), we were able to capture both overall self-reported habitual PA levels (which included a diverse array of activities), as well as more granular data on patterns of objective daily PA behaviors. Our use of multiple glaucoma-related outcomes allowed us to identify potentially important associations with glaucoma-related traits including IOP and measures of inner retinal thickness. We used corneal-compensated IOP as the measure of IOP, which may be better reflective of true IOP and may be associated more strongly with glaucoma risk.

We conducted gene—environment interaction analyses to examine whether PA might have differential associations with glaucoma based on level of genetic risk for glaucoma, given increasing evidence that certain lifestyle factors may be evident only in those with higher genetic risk of glaucoma.^28^ We additionally conducted MR analyses that allowed us to assess whether any of the observed cross-sectional associations may be causal.

Our study is limited by its use of self-reported data. Data collected using questionnaires can be subject to recall, social desirability, and misclassification biases. Our study is limited further by its use of self-reported medications and PA, although this is ameliorated partially using accelerometry, which provides greater objectivity in PA measurement. The use of two groups (self-reported and accelerometer) led to occasional and modest discrepancies in significance for some associations, and this is likely because of differences in statistical power between groups.

The definition of glaucoma was not highly specific and mainly relied on participant reporting, and our results thus may have been susceptible to various biases related to outcome misclassification. For glaucoma status in particular, it is possible that remote past PA in fact may be associated with glaucoma risk and that a glaucoma diagnosis may have led to lower PA levels, potentially biasing results to the null. Furthermore, the identified associations could have been the result of unmeasured exposures that link PA and mGCIPL thickness. Individuals who exercise more frequently or at higher intensities may have better overall health status and may be less likely to have vascular conditions that may lead to retinal changes or optic neuropathies that can affect inner retinal thickness. Additionally, UK Biobank participants may not necessarily be representative of the general population. We also did not have data on peripapillary RNFL thickness, which is a more commonly used measurement of optic nerve health in the assessment of glaucoma. One limitation of our MR analyses is the significant amount of participant overlap, which may result in biased results in the presence of weak instrument variables. In a 2-sample setting, the direction of this potential bias is away from the null.^77^ Although this is a possible explanation for some of the significant findings, it suggests that the identified null associations are truly nonsignificant.

Our study did not identify any association between overall PA level or greater duration of time spent in various PA levels and glaucoma status. Our study provided support for a positive association between greater time spent in habitual PA and thicker mGCIPL. Despite the well-documented acute reduction in IOP that occurs after an episode of PA, we found no evidence that PA or habitually high levels of PA are associated with long-term lower IOP in the general population.

## Supplementary Material

Supplement 1

Supplement 2

Supplement 3

Supplement 4

Supplement 6

Supplement 5

Supplement 7

## Figures and Tables

**Table 1.** Participant Characteristics by Cohort

	International Physical Activity Questionnaire-Derived Physical Activity			Accelerometry-Derived Physical Activity		
Characteristic	*Intraocular* *Pressure*	*OCT*	*Glaucoma*	*Intraocular* *Pressure*	*OCT*	*Glaucoma*
No. of patients	94 206	36 274	86 803	27 777	9 991	23 556
Age (yrs)	56.5 ± 8.0	56.2 ± 8.2	56.6 ± 8.1	56.5 ± 7.8	56.4 ± 7.9	56.5 ± 7.9
Sex						
Male	45 009 (48.0)	17 627 (48.6)	41 378 (47.7)	12 259 (44.1)	4 445 (44.5)	10 231 (43.4)
Female	48 739 (52.0)	18 647 (51.4)	45 425 (52.3)	15 518 (55.9)	5 546 (55.5)	13 325 (56.7)
Ethnicity						
White	85 962 (91.3)	33 180 (91.5)	78 568 (90.6)	26 377 (95.0)	9 459 (94.7)	22 230 (94.4)
Non-White	8 244 (8.7)	3 094 (8.5)	8235 (9.5)	1 400 (5.0)	532 (5.3)	1 326 (5.6)
Townsend deprivation index	−1.2 ± 2.9	−1.1 ± 2.9	−1.1 ± 3.0	−1.4 ± 2.8	−1.3 ± 2.8	−1.3 ± 2.8
Body mass index, kg/m^2^	27.2 ± 4.4	27.2 ± 4.4	27.2 ± 4.5	26.7 ± 4.3	26.7 ± 4.4	26.8 ± 4.4
Smoking status						
Never	52 579 (55.8)	19 870 (54.8)	48 195 (55.5)	15 906 (57.9)	5615 (56.2)	13 401 (56.9)
Former	32 836 (34.9)	12 882 (35.5)	30 232 (34.8)	9977 (36.0)	3705 (37.1)	8527 (36.2)
Current	8791 (9.3)	3522 (9.7)	8376 (9.7)	1894 (6.8)	671 (6.7)	1628 (6.9)
Alcohol status						
Never	4190 (4.5)	1491 (4.1)	4061 (4.7)	841 (3.0)	316 (3.2)	50 (3.2)
Former	3196 (3.4)	1266 (3.5)	3071 (3.5)	760 (2.7)	306 (3.1)	670 (2.8)
Current	86 820 (92.2)	33 517 (92.4)	79 671 (91.2)	26 176 (94.2)	9369 (93.8)	22 136 (93.4)
Diabetes status						
No	89 322 (94.8)	33 363 (95.2)	82 108 (94.6)	22 727 (96.4)	9369 (96.5)	22 662 (96.2)
Yes	4884 (5.2)	1674 (4.8)	4695 (5.4)	847 (3.6)	352 (3.5)	894 (3.8)
Systolic blood pressure (mmHg)	137.0 ± 18.3	136.7 ± 18.3	137.0 ± 18.3	136.3 ± 18.0	136.2 ± 18.2	136.3 ± 18.1
Spherical equivalent (D)	−0.39 ± 2.7	−0.03 ± 1.9	−0.39 ± 2.7	−0.54 ± 2.8	−0.14 ± 2.0	−0.59 ± 2.8
Height (cm)	169.1 ± 9.2	169.4 ± 9.2	169.0 ± 9.2	169.2 ± 9.0	169.4 ± 9.0	169.1 ± 9.0
IOP (mmHg)	16.0 ± 3.3	—	—	16.1 (3.3)	—	—
Macular RNFL thickness (μm)	—	28.9 ± 3.8	—	—	29.2 ± 3.8	—
Macular GCIPL thickness (μm)	—	75.2 ± 5.2	—	—	75.3 ± 5.2	—
Glaucoma status	—	—	1513 (1.7)	—	—	429 (1.8)

D = diopter; GCIPL = ganglion cell—inner plexiform layer; IOP = intraocular pressure; PA = physical activity; RNFL = retinal nerve fiber layer; — = not available.

Data are presented as no. (%) or mean ± standard deviation.

**Table 2.** Association between Physical Activity Levels and Glaucoma Status in the UK Biobank

Description	International Physical Activity Questionnaire-Derived Physical Activity		Accelerometry-Derived Physical Activity	
	Odds Ratio* (95% *Confidence Interval)*	P *Value*	*Odds Ratio*† *(95% Confidence Interval)*	P *Value*
Overall PA level				
Low	Reference	—	—	—
Moderate	0.87 (0.76—1.01)	0.06	—	—
High	0.97 (0.84—1.11)	0.63	—	—
P value (trend)		0.93		—
Activity level (per 30-min‡ increase in given activity level)				
Sedentary	1.01 (0.99—1.03)	0.47	1.00 (0.99—1.00)	0.98
Quartile 1	Reference	—	Reference	—
Quartile 2	0.99 (0.86—1.15)	0.86	0.97 (0.73—1.29)	0.81
Quartile 3	1.10 (0.95—1.27)	0.95	0.79 (0.59—1.07)	0.13
Quartile 4	1.01 (0.86—1.18)	0.86	1.10 (0.84—1.46)	0.48
*P* value (trend)		0.56		0.67
Low or light	1.00 (0.99—1.00)	0.26	1.00 (0.99—1.00)	0.26
Quartile 1	Reference	—	Reference	—
Quartile 2	1.00 (0.88—1.15)	0.96	**0.72 (0.55**—**0.95)**	0.019
Quartile 3	0.99 (0.85—1.15)	0.87	**0.69 (0.52**—**0.91)**	0.008
Quartile 4	1.11 (0.95—1.30)	0.21	0.94 (0.72—1.22)	0.63
*P* value (trend)		0.30		0.54
Moderate	1.00 (0.99—1.00)	0.94	1.00 (0.98—1.00)	0.42
Quartile 1	Reference	—	Reference	—
Quartile 2	0.97 (0.84—1.12)	0.67	**0.69 (0.52**—**0.90)**	0.007
Quartile 3	1.03 (0.89—1.19)	0.72	**0.73 (0.55**—**0.96)**	0.024
Quartile 4	1.00 (0.87—1.15)	0.99	0.99 (0.75—1.30)	0.92
*P* value (trend)		0.81		0.75
Vigorous	1.00 (0.99—1.00)	0.19	1.00 (0.99—1.00)	0.24
Quartile 1	Reference	—	Reference	—
Quartile 2	0.93 (0.80—1.09)	0.38	1.02 (0.79—1.32)	0.89
Quartile 3	**0.87 (0.76**—**0.99)**	0.037	0.90 (0.68—1.19)	0.47
Quartile 4	0.91 (0.79—1.05)	0.21	0.84 (0.62—1.14)	0.28
*P* value (trend)		0.07		0.21
Total PA	1.00 (0.99,1.00)	0.91	—	—
Quartile 1	Reference	—	—	—
Quartile 2	0.89 (0.77—1.03)	0.13	—	—
Quartile 3	0.95 (0.82—1.10)	0.49	—	—
Quartile 4	1.02 (0.88—1.18)	0.79	—	—
*P* value (trend)		0.58		—

PA = physical activity; — = not available. Boldface represents data that is statistically significant at *P* < 0.05.

Prevalent glaucoma derived from self-reported glaucoma or from glaucoma laser therapy or glaucoma surgery at baseline assessment, or from International Classification of Diseases, Tenth Revision, coded diagnoses of either primary open-angle glaucoma or unspecified glaucoma before or up to 1 year after baseline.

* Multivariable odds ratio adjusted for age, sex, ethnicity, Townsend deprivation index, body mass index, systolic blood pressure, smoking status, alcohol status, diabetes status, spherical equivalent, and height.

† Multivariable odds ratio adjusted for age, sex, ethnicity, Townsend deprivation index, body mass index, systolic blood pressure, smoking status, alcohol status, diabetes status, spherical equivalent, season, and height.

‡ For activity level analyses, International Physical Activity Questionnaire PA analyses are reported per additional 30 metabolic equivalent of task minutes of that given level of activity per week. Quartile 1 represents the lowest quartile of time spent in that given PA level, and quartile 4 represents the highest quartile of time spent in that given PA level. For sedentary: quartile 1, 0—180 minutes; quartile 2, 181—270 minutes; quartile 3, 271—360 minutes; quartile 4, 361— 1440 minutes. For light: quartile 1, 0—297 metabolic equivalent of task minutes/week; quartile 2, 298—693 metabolic equivalent of task minutes/week; quartile 3, 694—1386 metabolic equivalent of task minutes/week; quartile 4, 1387—4158 metabolic equivalent of task minutes/week. For moderate: quartile 1, 0—120 minutes; quartile 2, 121—480 minutes; quartile 3, 481—1200 minutes; quartile 4, 1201—5040 minutes. For vigorous: quartile 1, 0—79 minutes; quartile 2, 80—240 minutes; quartile 3, 241—960 minutes; quartile 4, 960—10 080 minutes. For accelerometer-derived PA, analyses are reported per additional 30 minutes of given level of activity per week. Quartile 1 represents the lowest quartile of time spent in that level of PA as measured by accelerometer.

**Table 3.** Association between Physical Activity Levels and Intraocular Pressure in the UK Biobank

Description	International Physical Activity Questionnaire -Derived Physical Activity		Accelerometry-Derived Physical Activity	
	*β Coefficient (95% Confidence Interval)* *	P *Value*	*β Coefficient (95% Confidence Interval)* †	P *Value*
Overall PA level				
Low	Reference	—	—	—
Moderate	0.06 (0.004—0.120)	0.037	—	—
High	0.08 (0.02—0.14)	0.010	—	—
*P* value (trend)		0.017		—
Activity level (per 30-min increase in given activity level) ‡				
Sedentary	−0.02 (−0.03 to −0.01)	< 0.001	0.001 (−0.001 to 0.001)	0.11
Quartile 1	Reference		Reference	—
Quartile 2	0.05 (−0.003 to 0.108)	0.06	0.28 (−0.08 to 0.14)	0.60
Quartile 3	0.01 (−0.05 to 0.07)	0.68	0.04 (−0.06 to 0.15)	0.43
Quartile 4	−0.09 (−0.16 to −0.03)	0.003	0.12 (0.01—0.23)	0.034
P value (trend)		0.003		0.037
Low or light	−0.001 (−0.001 to 0.000)	0.68	−0.003 (−0.006 to −0.001)	0.022
Quartile 1	Reference	—	Reference	—
Quartile 2	0.08 (0.02—0.13)	0.005	0.03 (−0.07 to 0.14)	0.54
Quartile 3	0.01 (0.04—0.16)	0.001	−0.08 (−0.19 to 0.02)	0.12
Quartile 4	0.07 (0.01—0.13)	0.030	−0.08 (−0.19 to 0.03)	0.14
P value (trend)		0.014		0.036
Moderate	0.001 (0.001—0.001)	0.050	0.001 (−0.002 to 0.005)	0.49
Quartile 1	Reference	—	Reference	—
Quartile 2	0.04 (−0.01 to 0.10)	0.15	0.04 (−0.07 to 0.15)	0.51
Quartile 3	0.06 (−0.003 to 0.115)	0.06	−0.02 (−0.13 to 0.09)	0.77
Quartile 4	0.08 (0.02—0.14)	0.005	0.03 (−0.08 to 0.15)	0.56
P value (trend)		0.005		0.81
Vigorous	−0.001 (−0.001 to 0.001)	0.21	0.01 (−0.02 to 0.04)	0.52
Quartile 1	Reference		Reference	—
Quartile 2	0.07 (0.003—0.129)	0.039	0.03 (−0.08 to 0.13)	0.65
Quartile 3	0.04 (−0.01 to 0.09)	0.14	0.08 (−0.03 to 0.19)	0.15
Quartile 4	0.01 (−0.05 to 0.06)	0.30	0.12 (0.01—0.24)	0.035
P value (trend)		0.55		0.22
Total PA	0.000 (−0.001 to 0.000)	0.61	—	—
Quartile 1	Reference	—	—	—
Quartile 2	0.05 (−0.01 to 0.11)	0.10	—	—
Quartile 3	0.06 (0.004—0.120)	0.035	—	—
Quartile 4	0.06 (−0.003 to 0.114)	0.035	—	—
*P* value (trend)		0.056		—

MET = metabolic equivalent of task; PA = physical activity; — = not available

* β Coefficient represents intraocular pressure difference using multivariable-adjusted model adjusting for age, sex, ethnicity, Townsend deprivation index, body mass index, systolic blood pressure, smoking status, alcohol status, diabetes status, spherical equivalent, and height.

† β Coefficient represents intraocular pressure difference using multivariable-adjusted model adjusting for age, sex, ethnicity, Townsend deprivation index, body mass index, systolic blood pressure, smoking status, alcohol status, diabetes status, spherical equivalent, height, and season.

‡ For activity level analyses, International Physical Activity Questionnaire PA analyses are reported per additional 30 metabolic equivalent of task minutes of that given level of activity per week. Quartile 1 represents the lowest quartile of time spent in that given PA level and quartile 4 represents the highest quartile of time spent in that given PA level. For accelerometer-derived PA, analyses are reported per additional 30 minutes of given level of activity per week. Quartile 1 represents the lowest quartile of time spent in that level of PA as measured by accelerometer, quartile 1, < 822; quartile 2, 822—1786; quartile 3, 1787—3573; and quartile 4, ≥ 3576. Units of measurement for the quartiles represent minutes spent per week in given level of PA.

## Abbreviations and Acronyms:

CI: confidence interval
GWAS: genome-wide association study
HbA1c: glycated hemoglobin
HES: Hospital Episode Statistics
ICD: International Classification of Diseases
IOP: intraocular pressure
IPAQ: International Physical Activity Questionnaire
LST: leisure screen time
MET: metabolic equivalent of task
mGCIPL: macular ganglion cell—inner plexiform layer
MR: Mendelian randomization
mRNFL: macular retinal nerve fiber layer
MVPA: moderate to vigorous physical activity
PA: physical activity
PRS: polygenic risk score
RGC: retinal ganglion cell
SNP: single nucleotide polymorphism.
