# Supplementary material for: The Association of Physical Activity with Glaucoma and Related Traits in the UK Biobank
Source: Ophthalmology. Author manuscript; Available in PMC 2024 Mar 5. (PMC10913205; doi:10.1016/j.ophtha.2023.06.009)
Supplement: Supplement 1 [file NIHMS1965544-supplement-Supplement_1.pdf]

Supplementary Table 4. Association between physical activity and macular retinal nerve fiber layer thickness (mRNFL) in the UK Biobank

Description	IPAQ-derived PA		Accelerometry-derived PA	
	β (95% CI)[a]	*P*-value	β (95% CI)[b]	*P*-value
Overall PA level				
Low	Reference	–	–	–
Moderate	0.01 (-0.10, 0.12)	0.84	–	–
High	0.003 (-0.11, 0.11)	0.96	–	–
P(trend)		0.99		–
Activity Level (per30 min[c] increase in given activity level)				
Sedentary	**-0.02 (-0.034, -0.002)**	**0.027**	0.001 (-0.001, 0.003)	0.46
Quartile 1	Reference	–	Reference	–
Quartile 2	-0.02 (-0.12, 0.09)	0.79	0.07 (-0.14, 0.27)	0.52
Quartile 3	-0.10 (-0.21, 0.01)	0.08	-0.08 (-0.28, 0.13)	0.47
Quartile 4	**-0.12 (-0.239, -0.003)**	**0.044**	0.03 (-0.19, 0.24)	0.82
P(trend)		**0.017**		0.82
Low / Light	-0.001 (-0.001, 0.001)	0.57	-0.001 (-0.006, 0.005)	0.69
Quartile 1	Reference	–	Reference	–
Quartile 2	-0.02 (-0.12, 0.08)	0.68	-0.15 (-0.36, 0.06)	0.17
Quartile 3	-0.05 (-0.16, 0.06)	0.39	-0.13 (-0.34, 0.08)	0.23
Quartile 4	-0.06 (-0.18, 0.06)	0.31	-0.11 (-0.32, 0.10)	0.31
P(trend)		0.26		0.38
Moderate	0.001 (-0.000, 0.002)	0.23	0.006 (-0.002, 0.013)	0.13
Quartile 1	Reference	–	Reference	–
Quartile 2	0.07 (-0.04, 0.18)	0.20	0.04 (-0.17, 0.26)	0.71
Quartile 3	0.05 (-0.06, 0.16)	0.37	0.01 (-0.21, 0.23)	0.92
Quartile 4	0.09 (-0.22, 0.20)	0.12	0.09 (-0.13, 0.31)	0.43
P(trend)		0.17		0.50
Vigorous	0.001 (-0.001, 0.001)	0.75	-0.003 (-0.06, 0.05)	0.93
Quartile 1	Reference	–	Reference	–
Quartile 2	0.12 (-0.002, 0.235)	0.06	0.03 (-0.18, 0.24)	0.77
Quartile 3	-0.003 (-0.10, 0.09)	0.95	0.10 (-0.11, 0.32)	0.35
Quartile 4	0.04 (-0.07, 0.14)	0.51	0.02 (-0.21, 0.24)	0.90
P(trend)		0.31		0.76
Total PA	0.001 (-0.000, 0.001)	0.64	–	–
Quartile 1	Reference	–	–	–
Quartile 2	0.01 (-0.10, 0.12)	0.83	–	–
Quartile 3	0.07 (-0.04, 0.18)	0.24	–	–
Quartile 4	-0.001 (-0.11, 0.11)	0.99	–	–
P(trend)		0.79		–

Abbreviations: CI, confidence interval; IPAQ, International Physical Activity Questionnaire; MET, metabolic equivalent of task; PA, physical activity. (a) *B* coefficient for multivariable-adjusted model adjusting for age, sex, ethnicity, Townsend deprivation index, body mass index, systolic blood pressure, smoking status, alcohol status, diabetes status, spherical equivalent, and height. (b) *B* coefficient for multivariable-adjusted model adjusting for age, sex, ethnicity, Townsend deprivation index, body mass index, systolic blood pressure, smoking status, alcohol status, diabetes status, spherical equivalent, height and season. (c) For Activity Level analyses, IPAQ PA results are reported per additional 30 *MET* mins of that given level of activity per week. Quartile 1 represents the lowest quartile of time spent in that given PA level and Quartile 4 represents the highest quartile of time spent in that given PA level. For accelerometer-derived PA, results are reported per additional 30 minutes of given level of activity per week. Quartile 1 represents the lowest quartile of time spent in that level of PA as measured by accelerometer, Quartile 1 (<822), Quartile 2 (822-1786), Quartile 3 (1787-3573), Quartile 4 (≥3576)
