# Supplementary material for: The Association of Physical Activity with Glaucoma and Related Traits in the UK Biobank
Source: Ophthalmology. Author manuscript; Available in PMC 2024 Mar 5. (PMC10913205; doi:10.1016/j.ophtha.2023.06.009)
Supplement: Supplement 3 [file NIHMS1965544-supplement-Supplement_3.pdf]

Supplementary Table 6 - Association of accelerometer-derived physical activity with intraocular pressure, macular retinal nerve fiber layer thickness and macular ganglion cell inner plexiform layer thickness, additionally adjusted for caffeine use

Difference in IOP (mmHg)			
Sedentary	<0.001	(-0.00002, 0.0001)	0.25
Low	-0.003	(-0.006, -0.0001)	**0.039**
Moderate	0.002	(-0.002, 0.006)	0.39
Vigorous	0.007	(-0.026, 0.039)	0.69
Difference in mRNFL thickness			
Sedentary	0.001	(-0.002, 0.004)	0.41
Low	-0.002	(-0.008, 0.003)	0.44
Moderate	0.007	(-0.001, 0.015)	0.09
Vigorous	0.01	(-0.05, 0.07)	0.76
Difference in mGCIPL thickness			
Sedentary	-0.001	(-0.004, 0.004)	0.77
Low	0.005	(-0.002, 0.013)	0.16
Moderate	0.02	(0.01, 0.03)	**<0.001**
Vigorous	0.10	(0.02, 0.18)	**0.012**

Abbreviations: IOP, intraocular pressure; mRNFL, macular retinal nerve fiber layer; mGCIPL, macular ganglion cell inner plexiform layer. Beta coefficients reported from multivariable-adjusted models adjusting for age, sex, ethnicity, Townsend deprivation index, body mass index, systolic blood pressure, smoking status, alcohol status, diabetes status, spherical equivalent, height, caffeine intake and season.
