# Supplementary material for: The Association of Physical Activity with Glaucoma and Related Traits in the UK Biobank
Source: Ophthalmology. Author manuscript; Available in PMC 2024 Mar 5. (PMC10913205; doi:10.1016/j.ophtha.2023.06.009)
Supplement: Supplement 4 [file NIHMS1965544-supplement-Supplement_4.pdf]

Supplementary Table 7 – Association of self-reported and accelerometry-derived physical activity with difference in inner retinal measurement thickness (additionally adjusted for Alzheimer's disease, Parkinsons Disease, and Multiple Sclerosis status)

	**Difference in inner retinal thickness (μm)**			
	mRNFL		mGCIPL	
	Coefficient (95% CI)	*P*-value	Coefficient (95% CI)	*P*-value
Self-reported PA (IPAQ)[a]				
Overall PA level				
Low	Reference		Reference	
Moderate	0.11 (-0.12, 0.34)	0.33	0.05 (-0.25, 0.36)	0.73
High	0.10 (-0.13, 0.33)	0.40	0.18 (-0.13, 0.49)	0.24
P value for trend		0.51		0.19
Activity level (per additional 30 MET mins increase)				
Sedentary	-0.01 (-0.05, 0.02)	0.41	**-0.06 (-0.10, -0.02)**	**0.006**
Low	0.002 (-0.001, 0.004)	0.16	0.003 (-0.0001, 0.0060)	0.06
Moderate	**0.002 (0.0003, 0.004)**	**0.022**	**0.003 (0.00001, 0.005)**	**0.049**
Vigorous	-0.001 (-0.003, 0.001)	0.35	0.001 (-0.002, 0.003)	0.65
Total	0.001 (-0.0003, 0.0015)	0.22	0.001 (-0.0001, 0.0020)	0.06
Accelerometry-derived PA[b]				
Sedentary	0.00003 (-0.0001, 0.001)	0.44	-0.00001 (-0.0010, 0.0001)	0.79
Low	-0.001 (-0.006, 0.004)	0.63	0.004 (-0.003, 0.010)	0.23
Moderate	0.005 (-0.002, 0.012)	0.17	**0.02 (0.01, 0.03)**	**<0.001**
Vigorous	-0.004 (-0.06, 0.05)	0.88	**0.10 (0.03, 0.18)**	**0.007**

Abbreviations: IOP, intraocular pressure; mRNFL, macular retinal nerve fiber layer; mGCIPL, macular ganglion cell inner plexiform layer. Beta coefficients reported per additional 30 MET mins / week spent in that level of activity. (a) Beta coefficients reported from multivariable-adjusted model adjusting for age, sex, ethnicity, Townsend deprivation index, body mass index, systolic blood pressure, smoking status, alcohol status, diabetes status, spherical equivalent, height and caffeine intake. (b) Beta coefficients for analyses with accelerometry-derived PA are reported from multivariable-adjusted model adjusting for age, sex, ethnicity, Townsend deprivation index, body mass index, systolic blood pressure, smoking status, alcohol status, diabetes status, spherical equivalent, height, season and caffeine intake.
