# Supplementary material for: The Association of Physical Activity with Glaucoma and Related Traits in the UK Biobank
Source: Ophthalmology. Author manuscript; Available in PMC 2024 Mar 5. (PMC10913205; doi:10.1016/j.ophtha.2023.06.009)
Supplement: Supplement 6 [file NIHMS1965544-supplement-Supplement_6.pdf]

The association of physical activity with glaucoma and related traits in the UK Biobank

APPENDIX 1: OPTICAL COHERENCE TOMOGRAPHY DATA ACQUISITION AND CLEANING

OCT data

As part of the UK Biobank Eye and Vision Consortium, 67 321 individuals underwent macular spectral domain OCT (SD-OCT) imaging as part of their baseline examinations between 2009 and 2010.[1] The Topcon 3D OCT1000 Mark II was used to complete SD-OCT imaging in a dark room without pupil dilation. The 3-dimensional 6x6 mm^2 macular volume scan mode (512 A scans per B scan and 128 horizontal B scans in a raster pattern) was used for imaging. Both eyes were imaged starting with the right eye. The OCT images were stored as downloadable electronic files from a secure portal. Version 1.6.1.1 of the Topcon Advanced Boundary Segmentation (TABS) algorithm was used to delineate the inner and outer retinal surfaces. Quality control to exclude images of poor quality was described in detail previously.[2] We excluded scans with an image quality score (signal strength) less than 45. Additionally, several segmentation indicators were calculated that also identified poor scan quality or segmentation failures; we excluded the poorest 20% of images for each of these indicators. The inner limiting membrane indicator was a measure of the minimum localized edge strength around the inner limiting membrane boundary across the entire scan; this is useful for identifying blinks, scans that contain regions of severe signal fading, and segmentation errors. The validity count indicator is used to identify scans with a significant degree of clipping in the OCT scan's z-axis dimension. The motion indicators use both the nerve fiber layer and the full retinal thicknesses, from which Pearson correlations and absolute differences between the thickness data from each

set of consecutive B-scans are calculated. The lowest correlation and the highest absolute difference in a scan serve as the resulting indicator scores and identify blinks, eye motion artifacts, and segmentation failures. The image quality score and the aforementioned indicators usually are highly correlated. We used average thickness parameters derived from the macula 6 grid. Participant-level macular retinal nerve fiber layer (mRNFL), and macular ganglion cell-inner plexiform layer (mGCIPL) thicknesses (in micrometers) were calculated as the mean of right and left eye values for each participant with good-quality images available for both eyes. If data were available only for 1 eye, we considered that value for the participant.[1]

References

1. Chua SYL, Thomas D, Allen N, et al. Cohort profile: Design and methods in the eye and vision consortium of UK Biobank. *BMJ Open*. 2019;9e025077.

2. Khawaja AP, Chua S, Hysi P, et al. Comparison of Associations with Different Macular Inner Retinal Thickness Parameters in a Large Cohort: The UK Biobank. *Ophthalmology*. 2020;127(1):62-71. doi:10.1016/j.ophtha.2019.08.015
