# Supplementary material for: The Association of Physical Activity with Glaucoma and Related Traits in the UK Biobank
Source: Ophthalmology. Author manuscript; Available in PMC 2024 Mar 5. (PMC10913205; doi:10.1016/j.ophtha.2023.06.009)
Supplement: Supplement 5 [file NIHMS1965544-supplement-Supplement_5.pdf]

Supplementary Table 8. Association between physical activity and macular ganglion cell-inner plexiform layer thickness in the UK Biobank, additionally adjusted for glycated hemoglobin (HbA1c)

Description	IPAQ-derived PA (n = 32 815)		Accelerometry-derived PA (n = 9 106)	
	Coefficient (95% CI)[a]	*P*-value	Coefficient (95% CI)[b]	*P*-value
Overall PA level				
Low	Ref		–	–
Moderate	0.14 (-0.01, 0.30)	0.06	–	–
High	**0.20 (0.04, 0.35)**	**0.012**	–	–
P(trend)		**0.017**		–
Activity Level (per 30 min[c] increase in given activity level)				
Sedentary	**-0.04 (-0.06, -0.01)**	**0.001**	-0.00002 (-0.0001, 0.0001)	0.73
Quartile 1	Reference		Reference	–
Quartile 2	-0.08 (-0.22, 0.07)	0.31	0.02 (-0.27, 0.30)	0.92
Quartile 3	-0.15 (-0.30, 0.01)	0.06	-0.11 (-0.40, 0.18)	0.45
Quartile 4	**-0.20 (-0.35, -0.03)**	**0.021**	-0.23 (-0.52, 0.07)	0.14
P(trend)		**0.013**		0.09
Low / Light	**0.003 (0.001, 0.004)**	**0.001**	0.004 (-0.003, 0.011)	0.29
Quartile 1	Reference		Reference	–
Quartile 2	0.001 (-0.14, 0.14)	0.99	0.05 (-0.24, 0.34)	0.74
Quartile 3	0.07 (-0.08, 0.23)	0.35	0.14 (-0.14, 0.43)	0.33
Quartile 4	**0.26 (0.09, 0.42)**	**0.002**	0.07 (-0.23, 0.36)	0.66
P(trend)		**0.002**		0.54
Moderate	0.001 (-0.001, 0.003)	0.06	**0.02 (0.01, 0.03)**	**<0.001**
Quartile 1	Reference		Reference	–
Quartile 2	**0.21 (0.06, 0.36)**	**0.005**	**0.45 (0.15, 0.74)**	**0.003**
Quartile 3	**0.16 (0.001, 0.312)**	**0.048**	**0.55 (0.26, 0.85)**	**<0.001**
Quartile 4	**0.21 (0.06, 0.37)**	**0.006**	**0.56 (0.25, 0.86)**	**<0.001**
P(trend)		**0.020**		**<0.001**
Vigorous	0.001 (-0.001, 0.003)	0.052	**0.11 (0.03, 0.19)**	**0.006**
Quartile 1	Reference		Reference	–
Quartile 2	**0.20 (0.03, 0.36)**	**0.018**	0.14 (-0.16, 0.43)	0.36
Quartile 3	0.12 (-0.02, 0.26)	0.088	**0.31 (0.01 0.60)**	**0.041**
Quartile 4	**0.15 (0.003, 0.294)**	**0.045**	**0.38 (0.08, 0.69)**	**0.014**
P(trend)		**0.040**		**0.008**
Total PA	**0.001 (0.001, 0.002)**	**0.002**	–	–
Quartile 1	Reference		–	–
Quartile 2	0.06 (-0.09, 0.21)	0.44	–	–
Quartile 3	**0.24 (0.09, 0.40)**	**0.002**	–	–
Quartile 4	**0.21 (0.05, 0.36)**	**0.008**	–	–
P(trend)		**0.001**		–

Abbreviations: CI, confidence interval; IPAQ, International Physical Activity Questionnaire; MET, metabolic equivalent of task; PA, physical activity, HbA1c hemoglobin A1c (glycated hemoglobin). (a) Beta coefficients for multivariable-adjusted model adjusting for age, sex, ethnicity, Townsend deprivation index, body mass index, systolic blood pressure, smoking status, alcohol status, diabetes status, spherical equivalent, height and HbA1c. (b) Beta coefficients for multivariable-adjusted model adjusting for age, sex, ethnicity, Townsend deprivation index, body mass index, systolic blood pressure, smoking status, alcohol status, diabetes status, spherical equivalent, height, season and HbA1c. (c) For Activity Level analyses, IPAQ PA results are reported per additional 30 *MET* mins of that given level of activity per week. Quartile 1 represents the lowest quartile of time spent in that given PA level and Quartile 4 represents the highest quartile of time spent in that given PA level. For accelerometer-derived PA, results are reported per additional 30 minutes of given level of activity per week. Quartile 1 represents the lowest quartile of time spent in that level of PA as measured by accelerometer, Quartile 1 (<822), Quartile 2 (822-1786), Quartile 3 (1787-3573), Quartile 4 (≥3576)
