# Supplementary material for: The Association of Physical Activity with Glaucoma and Related Traits in the UK Biobank
Source: Ophthalmology. Author manuscript; Available in PMC 2024 Mar 5. (PMC10913205; doi:10.1016/j.ophtha.2023.06.009)

The association of physical activity with glaucoma and related traits in the UK Biobank

APPENDIX 2: MENDELIAN RANDOMIZATION ANALYSES

Study design

We conducted two-sample Mendelian randomization (MR) analyses to test for a causal association between two genetically-determined physical activity phenotypes and five glaucoma-related outcomes. MR is a technique which uses genetic variants as instrumental variables (IVs) to estimate the causal effect of an exposure on an outcome.[1] By leveraging the random allocation of alleles at conception, MR allows for estimates which are unbiased by residual confounding and reverse causation. Three assumptions need to be satisfied to ensure a valid IV: (1) the IV must be associated with the exposure; (2) the IV must not be associated with any confounder of the exposure-outcome relationship; and (3) the IV must affect the outcome only through the exposure of interest. If these assumptions are satisfied, an estimated casual effect can be calculated from the observed IV-exposure and IV-outcome associations.

Instrumental variable selection

We used published data from a recent large genome-wide association study (GWAS) meta-analysis (including the UK Biobank) of physical activity to guide construction of our instrumental variables.[2] The study identified 89 and 11 independent genetic variants associated (at $P<5x10^{-9}$) with 'leisure screen time' (LST) and 'moderate-to-vigorous physical activity' (MVPA), respectively. We included only significant single nucleotide polymorphisms (SNPs) from the primary meta-analyses of European ancestry participants (*n* up to 606,820 for LST and *n* up to 526,725 for MVPA). Genetic principal components, population stratification and participant relatedness were adjusted for in the original study. At loci with multiple genome-wide significant SNPs, we excluded those with linkage disequilibrium R^2>0.001 and within 10,000 kb, retaining only the SNP with the lowest *P*-value, using the 1000 Genomes Project European reference population.[3] Palindromic SNPs with minor allele frequency >0.42, or when allele frequencies were not reported, were excluded. Effect alleles were harmonized across exposure and outcome datasets. Full details of these SNPs and their associations with glaucoma and related traits are available in **Supplementary Table 1**.

Outcome data sources

We utilized publicly-available summary statistics from large GWAS and GWAS meta-analyses for five glaucoma-related outcomes in participants of European ancestry. These included intraocular pressure

(IOP) (*n*=139,555),[4] macular retinal nerve fiber layer thickness (mRNFL) (*n*=31,434),[5] macular ganglion cell-inner plexiform layer thickness (mGCIPL) (*n*=31,434),[5] vertical cup-disc ratio (vCDR) (*n*=111,724),[6] and primary open-angle glaucoma (POAG) (*n*=216,257).[7] All outcome data sources included UK Biobank participants in their analyses.

Statistical analyses

The main MR analyses were performed using a multiplicative random-effects inverse-variance weighted (IVW) method.[9] This method provides precise and efficient estimates but is sensitive to invalid IVs and pleiotropy.[10] We therefore conducted sensitivity analyses using three alternative MR methods: weighted median,[11] MR-Egger[12] and MR pleiotropy residual sum and outlier (MR-PRESSO).[13] Each method makes different assumptions about the nature of pleiotropy and consistent estimates across methods strengthens causal inferences.[14] The weighted median method gives consistent estimates if the majority of IVs are valid, while the weighted mode method assumes that a plurality of IVs are valid. The MR-Egger and MR-PRESSO methods can test and correct for directional pleiotropy.

We assessed for heterogeneity with the I^2 and Cochran's Q statistics in the IVW model and with Rucker's Q' statistic in MR-Egger regression. The I^2_{GX} statistic is an indicator of expected relative bias (or dilution) of the MR-Egger causal estimate.[15] In MR-Egger regression, a significant difference of the intercept from zero is evidence for average directional horizontal pleiotropy.[12] The MR-PRESSO global test evaluates for horizontal pleiotropy, the outlier test detects specific SNP outliers, and the distortion test evaluates whether there is a significant difference in the causal estimate before and after adjusting for outliers.[13] Full results of these tests and statistics are available in **Supplementary Table 3**.

MR estimates are presented as unit change in the outcome per one standard deviation increase in the genetic instrument. We applied a conservative Bonferroni-corrected significance threshold of $P<0.005$ ($P<0.05/10$, to account for tests between two exposures and five outcomes). All analyses were performed in R version 4.1.1 (https://www.R-project.org) using the *MendelianRandomization* and *MRPRESSO* packages.

References

1. Davies NM, Holmes M V, Davey Smith G. Reading Mendelian randomisation studies: a guide, glossary, and checklist for clinicians. BMJ. 2018;362:k601.

2. Wang Z, Emmerich A, Pillon NJ, Moore T, Hemerich D, Cornelis MC, et al. Genome-wide association analyses of physical activity and sedentary behavior provide insights into underlying mechanisms and roles in disease prevention. Nat Genet. 2022;54(9):1332–44.

3. Auton A, Abecasis GR, Altshuler DM, Durbin RM, Abecasis GR, Bentley DR, et al. A global reference for human genetic variation. Nature. 2015;526(7571):68–74.

4. Khawaja AP, Cooke Bailey JN, Wareham NJ, Scott RA, Simcoe M, Igo RP, et al. Genome-wide analyses identify 68 new loci associated with intraocular pressure and improve risk prediction for primary open-angle glaucoma. Nat Genet. 2018;50(6):778–82.

5. Currant H, Hysi P, Fitzgerald TW, Gharahkhani P, Bonnemaijer PWM, Senabouth A, et al. Genetic variation affects morphological retinal phenotypes extracted from UK Biobank optical coherence tomography images. PLoS Genet. 2021;17(5):e1009497.

6. Han X, Steven K, Qassim A, Marshall HN, Bean C, Tremeer M, et al. Automated AI labeling of optic nerve head enables insights into cross-ancestry glaucoma risk and genetic discovery in >280,000 images from UKB and CLSA. Am J Hum Genet. 2021;108(7):1204–16.

7. Gharahkhani P, Jorgenson E, Hysi P, Khawaja AP, Pendergrass S, Han X, et al. Genome-wide meta-analysis identifies 127 open-angle glaucoma loci with consistent effect across ancestries. Nat Commun. 2021;12(1):1258.

8. Burgess S, Davies NM, Thompson SG. Bias due to participant overlap in two-sample Mendelian randomization. Genet Epidemiol. 2016;40(7):597–608.

9. Burgess S, Butterworth A, Thompson SG. Mendelian Randomization Analysis With Multiple Genetic Variants Using Summarized Data. Genet Epidemiol. 2013;37(7):658–65.

10. Burgess S, Bowden J, Fall T, Ingelsson E, Thompson SG. Sensitivity Analyses for Robust Causal Inference from Mendelian Randomization Analyses with Multiple Genetic Variants. Epidemiology. 2017;28(1):30–42.

11. Bowden J, Davey Smith G, Haycock PC, Burgess S. Consistent Estimation in Mendelian Randomization with Some Invalid Instruments Using a Weighted Median Estimator. Genet Epidemiol. 2016;40(4):304–14.

12. Bowden J, Davey Smith G, Burgess S. Mendelian randomization with invalid instruments: effect estimation and bias detection through Egger regression. Int J Epidemiol. 2015;44(2):512–25.

13. Verbanck M, Chen C-Y, Neale B, Do R. Detection of widespread horizontal pleiotropy in causal relationships inferred from Mendelian randomization between complex traits and diseases. Nat Genet. 2018;50(5):693–8.

14. Lawlor DA, Tilling K, Davey Smith G. Triangulation in aetiological epidemiology. Int J Epidemiol. 2016;45(6):1866–86.

15. Bowden J, Del Greco M F, Minelli C, Davey Smith G, Sheehan NA, Thompson JR. Assessing the suitability of summary data for two-sample Mendelian randomization analyses using MR-Egger regression: the role of the I2 statistic. Int J Epidemiol. 2016;45(6):1961–74.

Supplementary Table 1. Details of SNPs included in the physical activity instrumental variables and their association with glaucoma and related traits

SNP	Chr	BP	RA	EA	EAF	LST		IOP		mRNFL		mGCIPL		vCDR		POAG	
						Beta	SE	Beta	SE	Beta	SE	Beta	SE	Beta	SE	Beta	SE
rs3791033	1	44134077	T	C	0.33	-0.033	0.004	0.066	0.014	-0.024	0.038	0.010	0.050	0.001	0.001	0.007	0.014
rs10889193	1	61106174	A	C	0.45	-0.024	0.004	–	–	0.074	0.037	0.046	0.048	-0.001	0.001	-0.037	0.022
rs71658797	1	77967507	A	T	0.88	-0.037	0.006	–	–	-0.056	0.056	-0.136	0.073	–	–	–	–
rs12062845	1	98342685	A	C	0.78	-0.028	0.004	0.014	0.016	0.101	0.044	0.040	0.058	0.000	0.001	0.013	0.015
rs197439	1	112280990	G	A	0.60	-0.026	0.004	0.035	0.013	-0.012	0.037	-0.019	0.049	0.001	0.001	-0.009	0.013
rs6685030	1	171805284	G	A	0.48	-0.022	0.004	-0.002	0.013	0.053	0.036	-0.013	0.048	0.000	0.000	-0.026	0.013
rs62131183	2	45102896	G	A	0.05	-0.054	0.009	-0.029	0.032	0.138	0.086	0.070	0.114	0.001	0.002	-0.006	0.032
rs10189857	2	60713235	G	A	0.57	-0.027	0.004	0.025	0.013	0.007	0.037	-0.013	0.048	0.000	0.001	0.014	0.013
rs4303732	2	100830040	T	C	0.40	-0.027	0.004	-0.021	0.013	0.027	0.037	0.110	0.048	0.000	0.000	-0.025	0.013
rs62151809	2	104433256	T	C	0.55	-0.023	0.004	0.007	0.013	-0.015	0.037	0.016	0.048	0.000	0.001	0.012	0.013
rs114590429	2	166176789	A	C	0.88	-0.038	0.006	0.031	0.021	-0.071	0.056	0.018	0.074	0.001	0.001	0.013	0.020
rs12617870	2	193746283	T	G	0.46	-0.026	0.004	0.012	0.013	0.001	0.037	0.026	0.048	0.000	0.001	-0.008	0.013
rs36079846	2	215367159	C	T	0.52	-0.024	0.004	-0.014	0.013	0.033	0.036	0.007	0.048	0.000	0.000	-0.001	0.013
rs7615206	3	49937505	C	T	0.57	-0.035	0.004	-0.012	0.013	-0.026	0.037	-0.105	0.048	0.002	0.001	0.017	0.013
rs76267866	3	70540347	T	A	0.79	-0.030	0.005	–	–	-0.095	0.045	0.005	0.059	–	–	–	–
rs62244886	3	71587392	C	G	0.40	-0.024	0.004	–	–	-0.054	0.037	0.035	0.049	–	–	–	–
rs7430216	3	75201030	T	C	0.78	-0.025	0.004	0.027	0.016	-0.001	0.044	0.001	0.057	0.000	0.001	0.002	0.016
rs1375561	3	85658230	T	C	0.35	-0.023	0.004	0.024	0.014	0.041	0.038	0.037	0.050	0.000	0.000	0.009	0.014
rs9867121	3	114631548	C	A	0.18	-0.032	0.005	-0.043	0.017	0.077	0.047	0.015	0.061	0.000	0.001	-0.040	0.017
rs743699	4	3305116	G	A	0.74	-0.027	0.004	-0.010	0.015	-0.019	0.042	-0.008	0.055	-0.001	0.001	0.015	0.015
rs4416502	4	77030872	A	G	0.80	-0.029	0.005	-0.010	0.016	0.029	0.045	0.014	0.060	-0.001	0.001	-0.033	0.016
rs13107325	4	103188709	T	C	0.93	-0.040	0.007	0.043	0.025	0.063	0.070	-0.020	0.091	0.003	0.001	0.025	0.026
rs262890	5	62930015	G	A	0.70	-0.034	0.004	0.009	0.014	0.009	0.040	0.047	0.052	0.000	0.000	0.026	0.014
rs249960	5	96164771	A	G	0.18	-0.030	0.005	-0.005	0.017	-0.029	0.047	-0.134	0.062	0.001	0.001	0.010	0.017
rs396321	5	112113735	C	T	0.51	-0.021	0.004	0.016	0.013	0.032	0.036	-0.020	0.048	0.003	0.001	0.028	0.013
rs2964252	5	152067929	G	A	0.32	-0.024	0.004	0.013	0.014	-0.010	0.039	-0.003	0.051	0.001	0.001	-0.019	0.014
rs1947066	5	161101615	A	G	0.20	-0.030	0.004	-0.008	0.016	0.000	0.045	0.014	0.060	0.000	0.000	-0.013	0.016
rs558134	6	12693454	C	T	0.38	-0.023	0.004	0.021	0.013	0.024	0.037	0.046	0.049	-0.001	0.001	0.012	0.013
rs6457816	6	35362848	C	T	0.93	-0.041	0.007	0.038	0.026	-0.062	0.073	-0.124	0.096	0.001	0.001	0.048	0.026
rs78394231	6	107649123	C	T	0.90	-0.038	0.007	-0.009	0.022	-0.029	0.060	-0.081	0.079	0.001	0.001	-0.011	0.022
rs58541850	6	166165563	A	G	0.94	-0.052	0.008	-0.048	0.029	0.088	0.079	0.180	0.103	-0.002	0.001	-0.028	0.031
rs2529484	7	111180444	C	G	0.65	-0.022	0.004	–	–	0.040	0.038	0.030	0.050	–	–	–	–
rs13235840	7	133505091	T	A	0.82	-0.031	0.005	–	–	-0.047	0.047	-0.095	0.062	–	–	–	–
rs17621391	7	140176596	T	C	0.26	-0.024	0.004	-0.034	0.015	-0.032	0.041	-0.056	0.054	0.000	0.000	-0.004	0.015
rs7821826	8	10769439	T	C	0.51	-0.021	0.004	0.010	0.013	0.039	0.036	0.079	0.048	0.001	0.001	0.011	0.014
rs12678836	8	92690148	A	C	0.58	-0.023	0.004	-0.003	0.013	0.010	0.037	0.012	0.048	0.000	0.001	0.023	0.013
rs2783992	9	1722044	C	T	0.54	-0.024	0.004	-0.001	0.013	0.000	0.036	0.013	0.048	-0.001	0.001	-0.031	0.013

rs34864022	9	22609110	G	A	0.93	-0.048	0.008	0.038	0.026	-0.116	0.073	-0.144	0.096	-0.002	0.002	-0.020	0.026
rs1999065	9	120514574	T	C	0.66	-0.025	0.004	0.009	0.014	0.026	0.038	0.080	0.051	0.000	0.000	-0.008	0.014
rs7358158	9	140251458	A	G	0.88	-0.037	0.006	-0.045	0.022	–	–	–	–	–	–	–	–
rs68049022	10	66407019	T	C	0.20	-0.031	0.005	-0.003	0.017	0.052	0.045	0.100	0.060	-0.002	0.001	-0.005	0.016
rs841020	10	125409953	T	C	0.81	-0.027	0.004	0.005	0.016	0.035	0.047	-0.014	0.061	-0.001	0.001	0.004	0.016
rs4483592	11	65990439	T	C	0.84	-0.036	0.005	-0.008	0.017	0.030	0.049	0.029	0.065	0.001	0.001	0.031	0.017
rs1391954	11	88575965	T	G	0.56	-0.025	0.004	0.059	0.015	-0.282	0.036	-0.180	0.048	0.000	0.000	0.035	0.013
rs3759344	12	6862646	A	G	0.89	-0.045	0.006	0.020	0.021	0.109	0.060	0.079	0.079	0.001	0.001	0.002	0.022
rs10772643	12	13415288	C	T	0.89	-0.039	0.006	-0.004	0.021	0.024	0.059	0.110	0.078	0.000	0.001	0.045	0.020
rs7969719	12	109883577	T	C	0.69	-0.027	0.004	0.035	0.014	-0.007	0.039	-0.013	0.051	0.001	0.001	0.037	0.014
rs9513416	13	99055774	A	G	0.16	-0.028	0.005	-0.006	0.018	0.016	0.050	0.140	0.065	0.001	0.001	-0.006	0.018
rs10400776	14	97326366	C	A	0.26	-0.026	0.004	-0.024	0.015	-0.006	0.041	0.048	0.054	0.000	0.000	-0.017	0.015
rs12324720	15	64092140	G	A	0.18	-0.027	0.005	0.041	0.017	0.037	0.048	0.131	0.063	-0.001	0.001	0.028	0.016
rs56151256	15	78024806	A	C	0.25	-0.029	0.004	0.003	0.015	-0.049	0.042	-0.133	0.055	0.000	0.001	0.013	0.015
rs11074658	16	10308335	C	T	0.59	-0.024	0.004	0.025	0.013	0.039	0.037	0.030	0.049	0.000	0.000	0.014	0.013
rs4889530	16	31065918	T	A	0.38	-0.025	0.004	–	–	-0.075	0.037	-0.023	0.049	–	–	–	–
rs1860337	17	60851559	C	T	0.60	-0.025	0.004	-0.032	0.013	-0.015	0.037	-0.011	0.048	0.001	0.000	0.004	0.013
rs73420302	17	77768068	G	C	0.18	-0.030	0.005	–	–	0.013	0.048	-0.046	0.063	–	–	–	–
rs12962050	18	35179808	G	A	0.65	-0.023	0.004	-0.001	0.013	0.038	0.038	0.031	0.050	0.001	0.001	0.002	0.014
rs2229383	19	10794630	G	T	0.63	-0.029	0.004	-0.034	0.013	-0.008	0.038	-0.192	0.049	0.001	0.000	0.001	0.014
rs6857	19	45392254	C	T	0.17	-0.037	0.005	-0.024	0.018	-0.097	0.049	-0.081	0.064	-0.001	0.001	-0.066	0.018
rs6010651	20	62418243	A	C	0.38	-0.024	0.004	0.001	0.013	0.064	0.038	0.036	0.049	-0.001	0.001	-0.001	0.014

SNP	Chr	BP	RA	EA	EAF	MVPA		IOP		mRNFL		mGCIPL		vCDR		POAG	
						Beta	SE	Beta	SE	Beta	SE	Beta	SE	Beta	SE	Beta	SE
rs1160545	2	100832269	C	T	0.40	0.025	0.004	-0.020	0.013	0.026	0.037	0.111	0.048	0.000	0.000	-0.026	0.013
rs7613360	3	49916710	T	C	0.60	0.025	0.004	-0.005	0.013	-0.021	0.037	-0.099	0.049	0.002	0.001	0.008	0.014
rs1691471	3	85011013	C	T	0.38	0.038	0.004	-0.046	0.013	-0.018	0.037	-0.025	0.049	0.038	0.001	-0.051	0.014
rs13201721	6	141799534	C	T	0.74	0.026	0.004	-0.026	0.015	0.003	0.041	0.140	0.054	0.026	0.001	0.035	0.015
rs1625595	11	66078129	T	C	0.53	0.021	0.003	0.021	0.013	-0.012	0.036	-0.006	0.047	0.021	0.001	0.029	0.013
rs385301	17	19806828	T	C	0.74	0.028	0.005	0.028	0.016	0.050	0.041	-0.012	0.054	0.028	0.001	-0.050	0.015

Abbreviations: BP, base position (build 37); Chr, chromosome; EA, effect allele; EAF, effect allele frequency; IOP, intraocular pressure; LST, leisure screen time; mGCIPL, macular ganglion cell-inner plexiform layer; mRNFL, macular retinal nerve fiber layer; MVPA, moderate-to-vigorous physical activity; POAG, primary open-angle glaucoma; RA, reference allele; SE, standard error; SNP, single-nucleotide polymorphism; vCDR, vertical cup-disc ratio.

Supplementary Table 2. Results of Mendelian randomization analyses

	IOP (mmHg)		mRNFL (µm)		mGCIPL (µm)		vCDR		POAG (%)	
	Beta (95% CI)	P-value	Beta (95% CI)	P-value	Beta (95% CI)	P-value	Beta (95% CI)	P-value	Odds ratio (95% CI)	P-value
**LST**										
IVW	-0.15 (-0.39, 0.09)	0.22	0.00 (-0.57, 0.57)	0.99	0.06 (-0.60, 0.72)	0.86	-0.01 (-0.01, 0.00)	0.12	0.92 (0.75, 1.13)	0.44
Weighted median	-0.01 (-0.25, 0.24)	0.97	-0.07 (-0.64, 0.51)	0.82	-0.42 (-1.18, 0.34)	0.28	0.00 (-0.01, 0.01)	0.61	0.87 (0.69, 1.10)	0.24
MR-Egger	0.34 (-0.81, 1.49)	0.56	0.48 (-2.38, 3.34)	0.74	1.56 (-1.73, 4.85)	0.35	0.00 (-0.04, 0.04)	0.91	1.02 (0.37, 2.85)	0.97
MR-PRESSO	-0.05 (-0.26, 0.16)	0.66	-0.20 (-0.61, 0.21)	0.34	-0.23 (-0.80, 0.33)	0.42	0.00 (-0.01, 0.00)	0.16	0.88 (0.72, 1.07)	0.20
**MVPA**										
IVW	-0.62 (-1.11, -0.12)	0.014	0.10 (-1.01, 1.20)	0.86	0.45 (-2.15, 3.06)	0.73	0.01 (-0.03, 0.04)	0.66	0.60 (0.21, 1.71)	0.34
Weighted median	-0.82 (-1.37, -0.26)	0.004	-0.25 (-1.63, 1.13)	0.72	-0.47 (-2.50, 1.56)	0.65	0.00 (-0.02, 0.01)	0.72	0.33 (0.18, 0.62)	0.001
MR-Egger	-3.07 (-5.11, -1.03)	0.003	-0.45 (-6.12, 5.22)	0.88	-2.61 (-17.21, 11.99)	0.73	-0.13 (-0.32, 0.06)	0.19	0.01 (0.00, 0.60)	0.028
MR-PRESSO	–	–	–	–	-0.25 (-2.66, 2.16)	0.85	0.03 (-0.02, 0.08)	0.50	0.68 (0.18, 2.54)	0.67

Abbreviations: IOP, intraocular pressure; LST, leisure screen time; mGCIPL, macular ganglion cell-inner plexiform layer; MR-Egger, Mendelian Randomization-Egger; mRNFL, macular retinal nerve fiber layer; MR-PRESSO, Mendelian Randomization-Pleiotropy Residual Sum and Outlier; MVPA, moderate-to-vigorous physical activity; POAG, primary open-angle glaucoma; vCDR, vertical cup-disc ratio.

Supplementary Table 3. Tests of heterogeneity, directional pleiotropy and regression dilution statistics for the physical activity instrumental variables

	IOP		mRNFL		mGCIPL		vCDR		POAG	
	Estimate	*P*-value	Estimate	*P*-value	Estimate	*P* -value	Estimate	*P* -value	Estimate	*P* -value
**Leisure screen time**										
**IVW**										
Cochran's *Q* statistic	128.2 (50)	<0.001	123.0 (57)	<0.001	95.6 (57)	0.001	102.9 (50)	<0.001	97.3 (50)	<0.001
I^2 statistic	61.0%	–	53.7%	–	40.4%	–	51.4%	–	48.6%	–
**MR-Egger**										
Rucker's *Q'* statistic	126.3 (49)	<0.001	122.8 (56)	<0.001	94.2 (56)	0.001	102.7 (49)	<0.001	97.3 (49)	<0.001
I^2_{GX} statistic	0.0%	–	0.0%	–	0.0%	–	81.1%	–	0.0%	–
Intercept	-0.01	0.39	-0.01	0.74	-0.04	0.36	0.00	0.72	0.00	0.84
**MR-PRESSO**										
Global test	–	<0.001	–	<0.001	–	<0.001	–	<0.001	–	<0.001
Number of outliers	2	–	1	–	2	–	1	–	1	–
Distortion test	-213.7	0.15	101.7%	0.33	124.9%	0.30	-31.0%	0.58	37.1%	0.72
**Moderate-to-vigorous physical activity**										
**IVW**										
Cochran's *Q* statistic	7.5 (5)	0.18	2.6 (5)	0.76	16.1 (5)	0.007	38.8 (5)	<0.001	34.5 (5)	<0.001
I^2 statistic	33.6%	–	0.0%	–	68.9%	–	87.1%	–	85.5%	–
**MR-Egger**										
Rucker's *Q'* statistic	1.7 (4)	0.78	2.6 (4)	0.63	15.4 (4)	0.004	26.1 (4)	<0.001	17.3 (4)	0.002
I^2_{GX} statistic	53.0%	–	50.9%	–	50.8%	–	84.8%	–	51.5%	–
Intercept	0.07	0.016	0.02	0.85	0.09	0.68	0.00	0.16	0.12	0.046
**MR-PRESSO**										
Global test	–	0.21	–	0.76	–	0.018	–	0.001	–	<0.001
Number of outliers	0	–	0	–	1	–	4	–	4	–
Distortion test	–	–	–	–	280.4%	0.16	-72.1%	<0.001	-30.8%	>0.99

Abbreviations: CI, confidence interval; IOP, intraocular pressure; IVW, inverse variance weighted; mGCIPL, macular ganglion cell-inner plexiform layer; MR-PRESSO, Mendelian Randomization-Pleiotropy Residual Sum and Outlier; MR-Egger, Mendelian Randomization-Egger; mRNFL, macular retinal nerve fiber layer; POAG, primary open-angle glaucoma; vCDR, vertical cup-disc ratio.

Supplementary Figure 1. Scatter plot of the SNP-LST (exposure) and SNP-IOP (outcome) association estimates for the leisure screen time instrumental variable

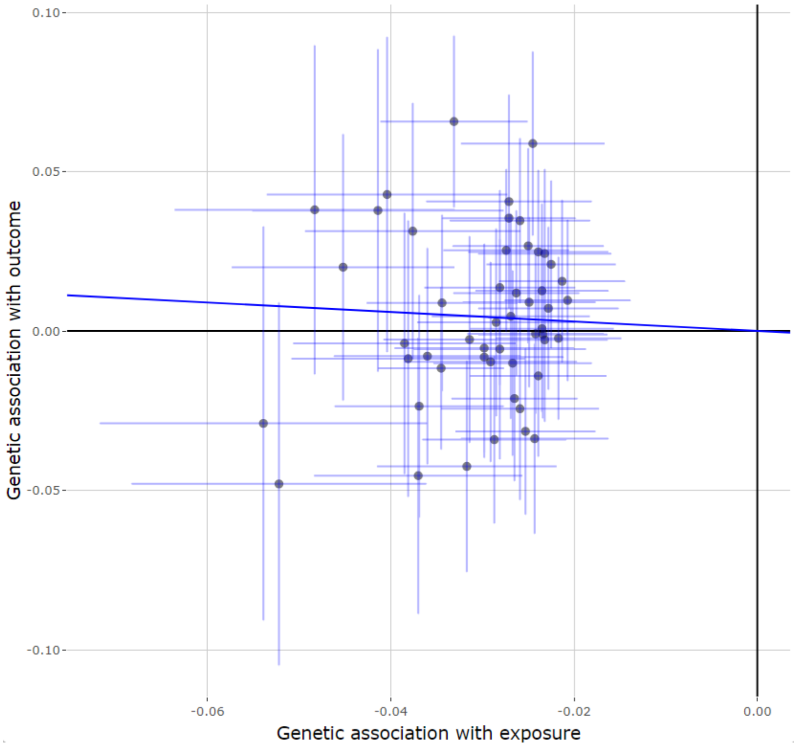

Supplementary Figure 2. Scatter plot of the SNP-LST (exposure) and SNP-mRNFL (outcome) association estimates for the leisure screen time instrumental variable

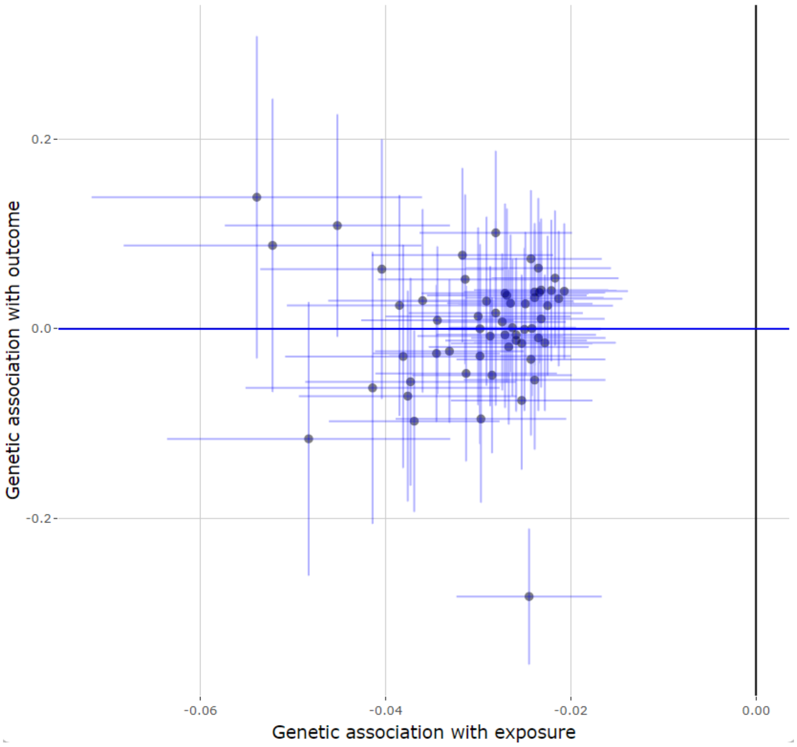

Supplementary Figure 3. Scatter plot of the SNP-LST (exposure) and SNP-mGCIPL (outcome) association estimates for the leisure screen time instrumental variable

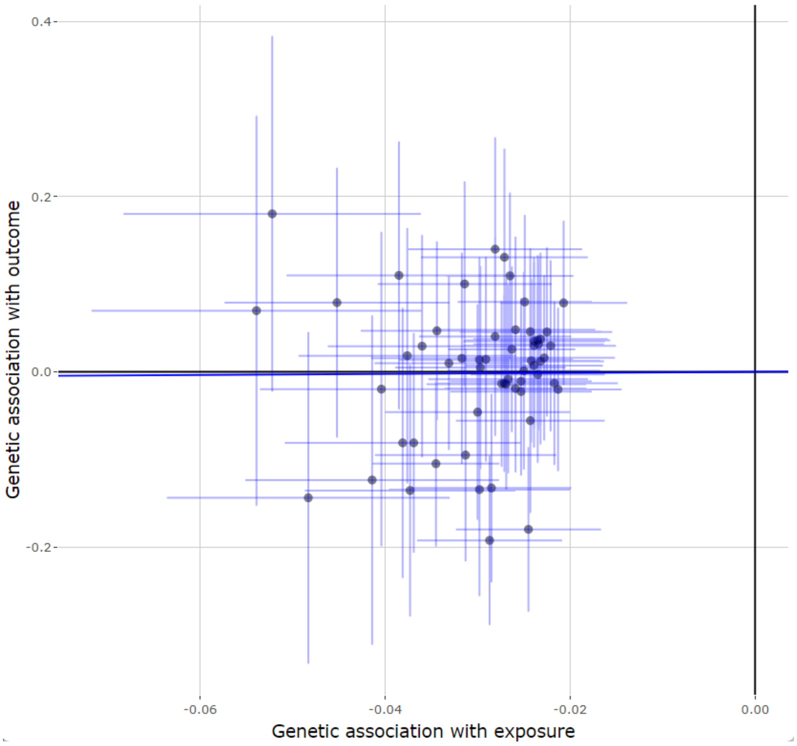

Supplementary Figure 4. Scatter plot of the SNP-LST (exposure) and SNP-vCDR (outcome) association estimates for the leisure screen time instrumental variable

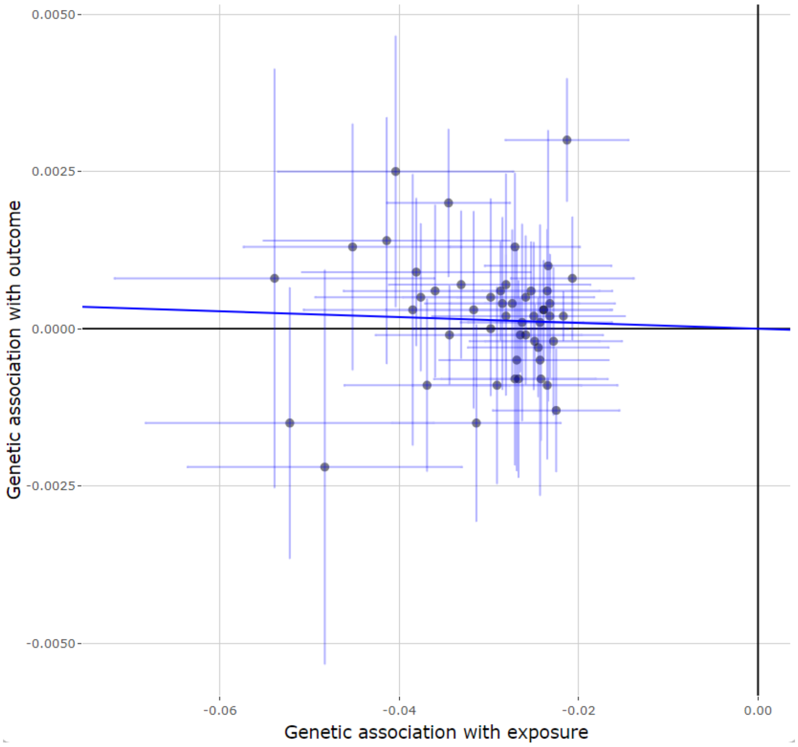

Supplementary Figure 5. Scatter plot of the SNP-LST (exposure) and SNP-POAG (outcome) association estimates for the leisure screen time instrumental variable

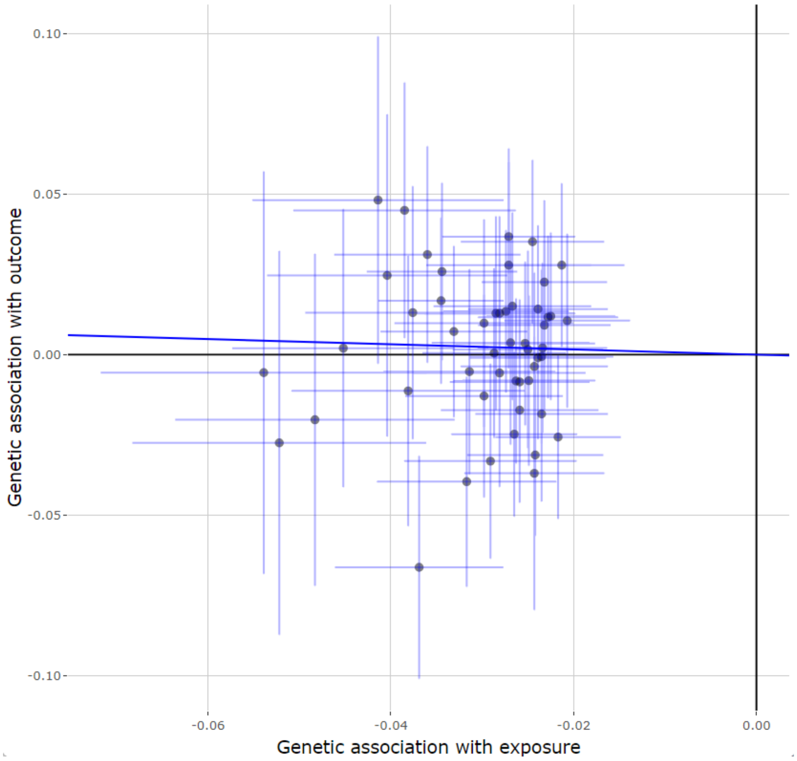

Supplementary Figure 6. Scatter plot of the SNP-MVPA (exposure) and SNP-IOP (outcome) association estimates for the moderate-to-vigorous physical activity instrumental variable

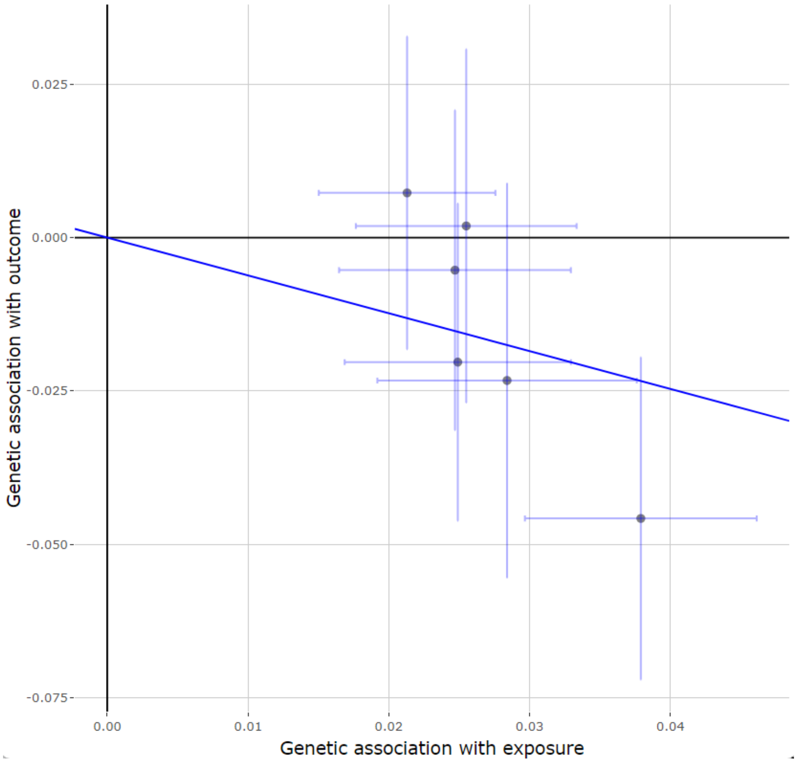

Supplementary Figure 7. Scatter plot of the SNP-MVPA (exposure) and SNP-mRNFL (outcome) association estimates for the moderate-to-vigorous physical activity instrumental variable

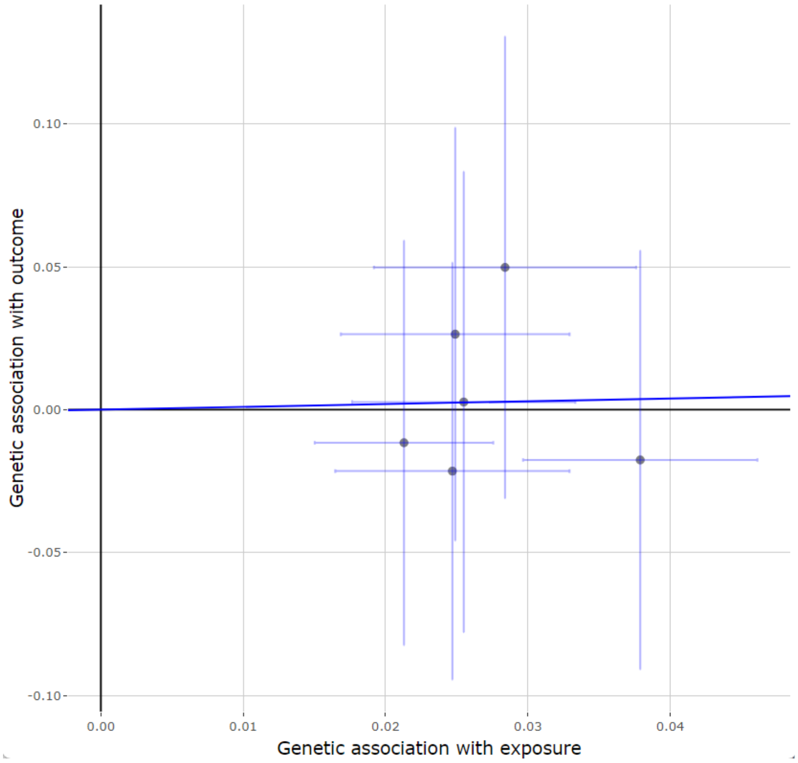

Supplementary Figure 8. Scatter plot of the SNP-MVPA (exposure) and SNP-mGCIPL (outcome) association estimates for the moderate-to-vigorous physical activity instrumental variable

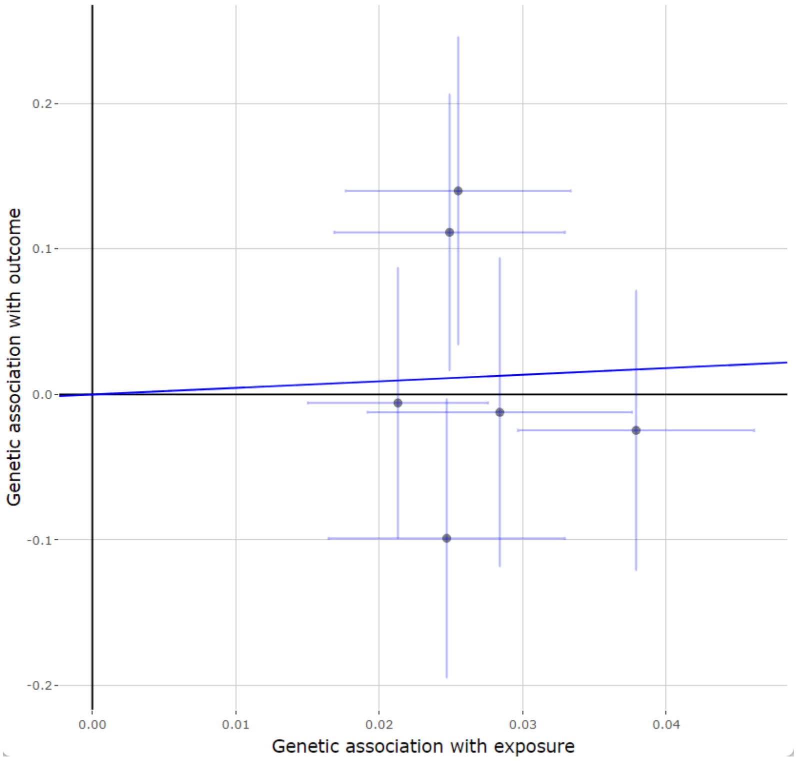

Supplementary Figure 9. Scatter plot of the SNP-MVPA (exposure) and SNP-vCDR (outcome) association estimates for the moderate-to-vigorous physical activity instrumental variable

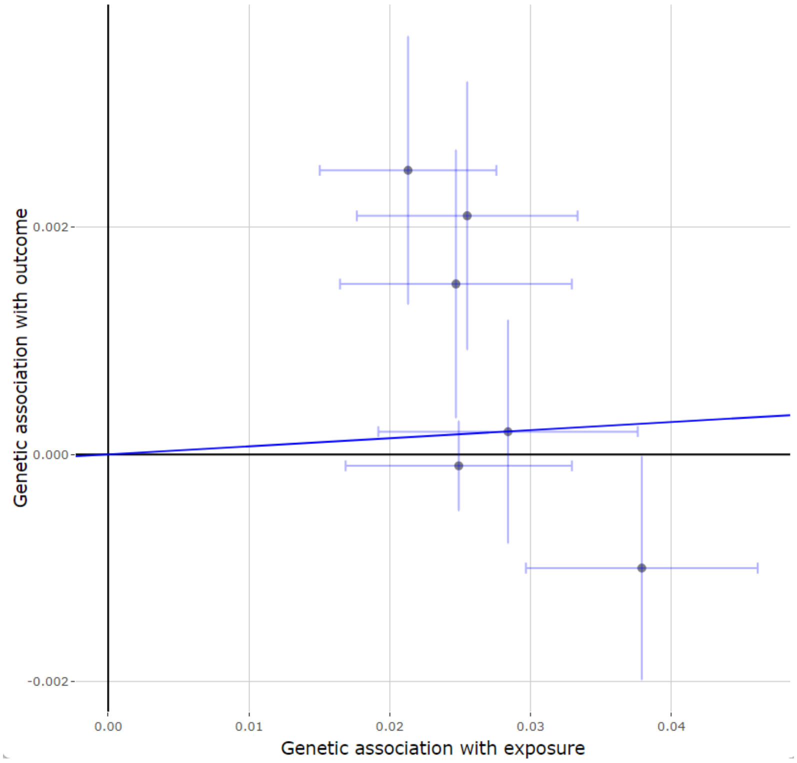

Supplementary Figure 10. Scatter plot of the SNP-MVPA (exposure) and SNP-POAG (outcome) association estimates for the moderate-to-vigorous physical activity instrumental variable

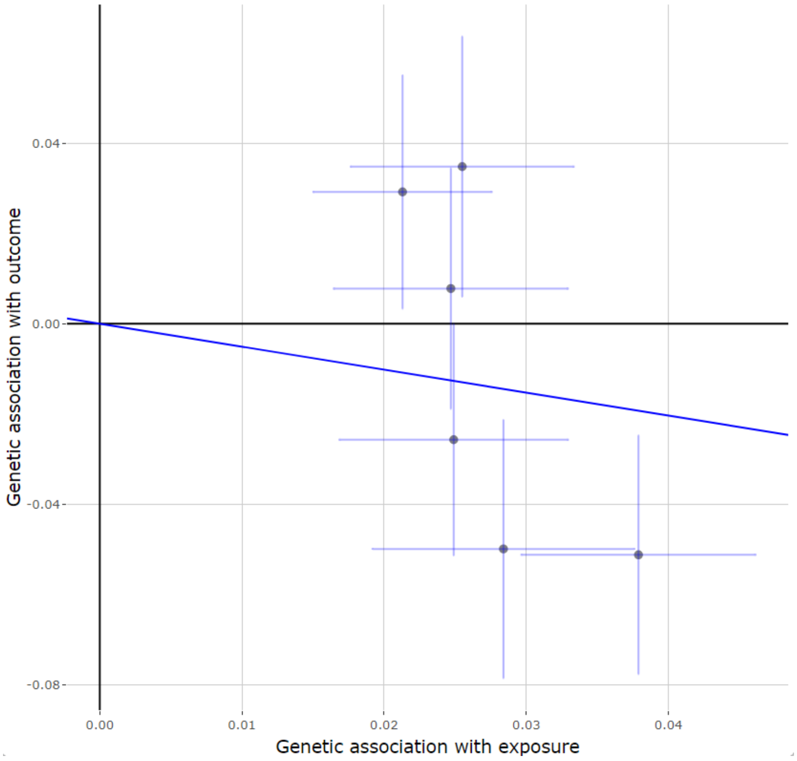

Supplement: Supplement 7 [file NIHMS1965544-supplement-Supplement_7.pdf]
